# Design a model to predict incomplete immunization among Ethiopian children using ensemble machine learning algorithms

**DOI:** 10.1038/s41598-025-31716-5

**Published:** 2025-12-12

**Authors:** Birku Getie Mihret, Betelhem Nega Belay, Jenberu Mekurianew Kelkay, Ayalew Melese Amare, Abibual Desalegn Tarekegn, Amare Alemu Asegu, Getnet Nega Belay, Asmamaw Mekure Engdayehu, Abebaw Agegne Engda, Yeshambel Asmare Mengist, Deje Sendek Anteneh, Seid Hassen Yesuf

**Affiliations:** 1 Department of Computer Science, College of Computational Sciences, Debark University, Debark, Ethiopia; 2 Department of Information Technology, South Gondar Zone Sport Department Office, Debre Tabor, Ethiopia; 3 Department of Computer Science, College of Informatics, University of Gondar, Gondar, Ethiopia; 4 Department of Public Health, College of Health Sciences, Debark University, Debark, Ethiopia; 5 Department of Information System, Guhalla TVET College, Gondar, Ethiopia; 6 Department of Computer Science, Gafat Institute of Technology, Debre Tabor University, Debre Tabor, Ethiopia; 7 Department of Veterinary Medicine, Farta Wereda Livestock and Fishery Resource Development Office, Debre Tabor, Ethiopia; 8 Department of Child Protection, Save the Children International, Debre Tabor, Ethiopia; 9 Department of Computer Science, Institute of Technology, Woldia University, Wolldia, Ethiopia; 10 Department of Health Informatics, Institute of Public Health, College of Medicine and Health Sciences, University of Gondar, Gondar, Ethiopia; 11 Department of Computer Science, Gafat Institute of Technology, Debre Tabor University, Debre Tabor, Ethiopia

**Keywords:** Ensemble learning, EDHS, Children, Immunization, Ethiopia, Computational biology and bioinformatics, Immunology, Endocrinology, Health care, Medical research, Molecular medicine, Mathematics and computing

## Abstract

Immunization is a cost-effective public health intervention globally, including in Ethiopia. However, the study focused on children aged 0–59 months and analyzed factors influencing incomplete immunization using ensemble machine learning techniques. A total of 16,394 EDHS datasets were used, with 80% for training and 20% for testing sets. Accordingly, the training set consisted of 13,115 samples, while the testing set contained 3,279 samples. Ensemble learning algorithms were employed, including Bagging methods (Bagging meta-estimator, Random Forest), Boosting methods (Gradient Boosting, XGBoost, LightGBM, AdaBoost, and CatBoost), and Voting ensembles combining both bagging and boosting models. Additionally, Stacking was performed using XGBoost and CatBoost as base models, with other machine learning algorithms such as Random Forest, K-Nearest Neighbors (KNN), Artificial Neural Networks (ANN), Support Vector Machines (SVM), and Logistic Regression as meta-models. All models were implemented using the Python programming language. On the tested data, bagging meta-estimator + XGBoost voting model executed the highest performance result of accuracy (95.94%), f1-score (95.89%), recall (94.81%), and precision (97.07%), for visualizing using a confusion matrix and AUC-ROC value of 96%, and the cross-validation score of 95.75% for its reliability. Also, the most influential factors for incomplete immunization include marital status, residence, and others. This study aims to identify key factors influencing immunization coverage among Ethiopian children under the age of five and improve with ensemble machine learning algorithms. The findings provide valuable insights for targeted interventions, supporting improved immunization practices and contributing to better child health outcomes.

## Introduction

 Immunization is one of the most cost-effective public health interventions, playing a critical role in reducing morbidity and mortality among children worldwide^1^. In Ethiopia, where preventable childhood diseases still contribute to significant health burdens, ensuring complete immunization coverage is vital. Despite the known benefits, incomplete immunization remains challenging, leaving many children vulnerable to diseases that could otherwise be prevented. The Expanded Program on Immunization (EPI) in Ethiopia aims to protect children from vaccine-preventable diseases (VPDs), such as cervical cancer, poliomyelitis, measles, rubella, parotitis, diphtheria, tetanus, pertussis, hepatitis A & B, bacterial pneumonia, rotavirus diarrheal diseases, and bacterial meningitis^2^. The World Health Organization (WHO) started a global vaccination program, the Expanded Program on Immunization (EPI), in 1974^3^.The program aimed to protect children from ten infectious and fatal childhood diseases, such as Diphtheria, Pertussis, Tetanus, Polio, Tuberculosis, Measles, Haemophilus influenzae meningitis, yellow fever, Hepatitis B, and Pneumococcal, by making the corresponding vaccines available to all. Even though the prevention of child mortality through immunization is one of the most cost-effective and widely applied public health interventions^4^. Even though those efforts in expanded programs of immunization, nearly one-fifth of children in developing countries miss out on basic immunizations. Moreover, many children who started vaccination fail to complete immunization, assess vaccination coverage, and identify gaps in immunization. It is important to distinguish between incomplete and complete immunization statuses. Incomplete immunization refers to cases where children have started the vaccination process but missed at least one dose of the recommended vaccines during the period from birth to 59 months (or 0 to 5 years)^1^. On the other hand, Complete Immunization is defined as when a child has received all the recommended vaccinations according to the immunization schedule. This includes one dose of Bacillus Calmette-Guérin(BCG), three doses of polio, three doses of diphtheria, pertussis, and tetanus(DPT), one dose of measles, three doses of pentavalent vaccine, three doses of pneumococcal conjugate vaccine(PCV), and two doses of rotavirus vaccine^5,6^.

Incomplete immunization can put children at greater risk of acquiring vaccine-preventable diseases. Every day, around 29,000 children under the age of five die in the world^7^.

Immunization remains a critical public health challenge in Ethiopia. According to the Ethiopian Ministry of Health, the 2011 DHS in Ethiopia, only 24.3% of children between 12 and 23 months of age were fully immunized^8^. Also, the 2016 Ethiopian EDHS report revealed that only 39% of children in Ethiopia received all recommended immunizations^6,]^^9^. Additionally, according to the 2019 EMDHS report, full immunization coverage has reached 43%, with a steady change over time^10^.

The study’s conclusions enable decision-makers in the relevant study area, such as policymakers and immunization program managers. Besides this, the lack of immunization is a significant cause of the death of children between the ages of five^11^. Even though immunization coverages are provided by healthcare workers at health facilities, healthcare access and utilization are important to consider when looking at predictors for complete immunization by using traditional statistical analysis techniques^12^.

As technology expands, machine learning provides an exciting opportunity in health care to improve the accuracy of diagnoses, personalize health care, and find solutions to specific problems in public health. In machine learning scenarios, it is useful for optimizing immunization, analyzing large datasets to identify patterns, predicting outcomes, and recommending targeted interventions. Also, the machine learning (ML) models were used to identify the factors of incomplete immunization based on demographic, socio-economic, and healthcare access data. Addressing incomplete immunization in Ethiopia requires a comprehensive understanding of the determinants influencing immunization uptake. Traditional approaches, while valuable, have limitations in identifying complex patterns and interactions between various factors. Machine learning offers a novel approach were studying the age of 12–35 months, and this study aims to fill the gap for all ages under five or 0–59 months by identifying the factors contributing to incomplete immunization^13^. This study aims to explore the application of ensemble machine learning algorithms to predict incomplete immunization among Ethiopian children. Specifically, it seeks to identify key determinants and their interactions that contribute to incomplete immunization within the basic age groups of the child, and to what extent ensemble learning techniques’ prediction models perform well.

This technique reduced model prediction error when the base models were diverse, independent, and used in the popular ensemble approaches^14^. To preprocess the 16,394 datasets for this study, procedures such as data reduction, data cleaning, data balancing (class weights, SMOTE, and Tomek Link), and the splitting phase should be followed, as others if available^15^. As the essential implementations using Python programming languages were used, ensemble supervised machine learning techniques such as Bagging (Bagging meta-estimator, Random Forest), Boosting (Gradient Boosting, XGBoost, LightGBM, AdaBoost, and CatBoost, and Voting (Bagging meta-estimator + Random Forest, GBoost + AdaBoost, LightGBM + CatBoost, Random Forest + LightGBM, Bagging ME + XBoost, AdaBoost + LightGBM, XGBoost + AdaBoost + LightGBM, LightGBM + GBoost + AdaBoost + XGBoost were used. Additionally, in stacking (with base models such as XGBoost and CatBoost), along with other machine learning algorithms like Random Forest, KNN, ANN, SVM, and a meta-model, Logistic Regression was also employed in the study^16^. These ensemble methods were also utilized to predict incomplete immunization among Ethiopian children and evaluate the training and testing data using different performance evaluation metrics, accuracy, recall, precision, and F1-score^17^. As a result, various predictive methods have assessed the factors contributing to incomplete immunization. To determine their effectiveness, the ensemble models were evaluated on the tested data. The model demonstrated impressive accuracy, reflecting its robust performance, and improved model abilities using hyperparameter tuning.

Therefore, this study aims to find contributions to the development of more effective strategies to increase immunization rates, ultimately improving child health outcomes in Ethiopia by investigating the factors associated with incomplete immunization.

## Literature review

### Overview of immunization

^12^^,18^ Immunization is a crucial element of primary health care and an undisputed human right. Measurable achievements of reducing morbidity and mortality associated with vaccine-preventable diseases (VPDs) have been documented, and a national immunization program was commenced in Ethiopia in 1980 G.C^19^.

Immunization has been promoted as a global strategy aimed at improving child survival. The World Health Organization strives to make immunization services available to everyone, everywhere, to save over 50 million lives by 2030^20^. Monitoring and identifying the factors contributing to the change in immunization coverage over time and across nations is imperative for continuing global success in increasing immunization coverage. The study examined the changes and factors that contributed to the change in full immunization coverage over time in Ethiopia (2000 to 2019)^21^. According to Ethiopian studies from 2005, 2011, and 2016 showed that the percentage of people who were not fully immunized was 74.6%, 71.4%, and 55.1%, respectively^13^. Further research is necessary to prioritize and promote childhood vaccination to guarantee the health and well-being of all Ethiopian children, since the alarmingly high rates of incomplete childhood immunization persist^22^.

### Determinant factors of incomplete immunization

Immunization is an effective public health intervention to reduce morbidity and mortality among children, and it will become more effective if children can receive the full course of recommended immunization doses. However, due to various reasons, many fail to complete the full course of immunization^23^.

### Socio-demographic factors

#### Educational status

Maternal education and husband education were revealed to be significant predictors of complete basic childhood immunization, according to multilevel analysis studies conducted in Ethiopia^10^^,22^. The findings showed that educated wives and husbands were more likely than uneducated wives and husbands to vaccinate their children throughout the whole range. It was also shown that immunization and the mother’s level of education were closely associated^21^. Prior research has demonstrated that women with education have a higher likelihood of immunizing their children than moms without education^6^.

#### Mothers’ age

Younger mothers had a higher likelihood than older mothers of skipping their child’s immunization sessions, according to a previous study^24^. Mothers over 35 had a 45% reduced likelihood of their child not receiving all recommended vaccines than mothers between the ages of 15 and 24 ^22^,^26^. The researchers concluded that, given their greater understanding of healthcare services, older mothers may appreciate their children’s full immunizations more than younger mothers^23,]^^26^.

#### Knowledge of immunization

The overall analysis of studies showed that maternal immunization knowledge is associated with incomplete vaccination. Knowledgeable women about vaccination were less likely to vaccinate their children compared to non-knowledgeable women incompletely^24,27,28^.

#### Maritial status

A prior study found that unmarried mothers had a higher chance of their children receiving fewer vaccinations than mothers who were married^24,26,28,29,30,31^.

#### Religion

Cultural and religious beliefs can significantly shape health behaviors, sometimes acting as barriers or facilitators to healthcare practices like vaccination^31^^,32^. By aligning healthcare messages with community values and religious beliefs, it is possible to foster a more supportive environment for immunization, helping to overcome potential resistance and improve vaccine uptake in various cultural contexts.

#### Occupation status

Previous studies have discovered a connection between parental occupation (unskilled workers) and incomplete childhood immunization^24,]^^25,]^^29^. Manual laborers who are poorly compensated and lack education may not have the time or resources to visit their local health facility to get vaccinated^28^.

#### Place of residence

Compared to mothers in urban regions, rural areas were more likely to give their children all recommended vaccinations^29^. Compared to children born to rural mothers, children born to urban mothers had a 1.8 times higher chance of receiving all recommended vaccinations^24^,^29^. This could be because mothers in rural areas have fewer other employment responsibilities than mothers in urban areas, which gives them more time to focus on their children’s health and ensure they obtain the recommended immunizations^23,26,32^.

#### Age of the child

^33^ Immunization schedules often follow age-specific guidelines, so delays or gaps in vaccination may occur as children grow older. Younger children are typically more likely to receive vaccines as part of standard health programs, while older children may be missed^22^.

#### Sex of the child

In some cultures or regions, there may be gender-based differences in access to healthcare services, including immunization^22^^32^,. Studies sometimes find differences in immunization rates between males and females due to social, cultural, or economic factors^30^.

### Socio-economic factors

#### Media exposure

The most effective way to enhance healthcare-seeking behavior in the community is through media exposure^31^. Exposure to the media is essential for disseminating information regarding childhood vaccines and encouraging modifications in behavior concerning these practices. Research findings indicate that mothers who do not have access to information are more likely to give their children an incomplete vaccine^23,25^.

#### Wealth index

Poverty in the home has an impact on childhood immunization. Children from impoverished households are more likely to have received insufficient vaccinations^29^. The children suffer from two types of deprivation as a result of their financial situation: they are deprived of nourishing food that could naturally strengthen their immune system, and they are also deprived of full immunization, which could protect them against diseases that can be prevented by vaccination^24,]^^27^.

### Reproductive history factors

#### Place of delivery

An Ethiopian study found a correlation between children’s full immunization status and the use of maternal health care services, including ANC and institutional delivery^34^. According to the systematic review and meta-analysis, women who gave birth at home were nearly 3 times more likely to have incompletely immunized children than women who delivered at health facilities^28^.

#### Antenatal care (ANC) follow-up and postnatal care (PNC) visits

Full childhood immunization rates were higher in mothers who utilized maternal health care services (ANC and PNC visits) than in mothers who did not^27^. Attending prenatal care increases the likelihood that women will obtain sufficient knowledge of routine infant immunization^24^. Pregnancy-related treatments also set up a woman for a positive likelihood of using healthcare services for herself and her offspring. In Ethiopia, research found that children whose mothers had received the Tetanus Toxoid (TT) vaccination and followed ANC during their most recent pregnancy were more likely to have had all of the necessary vaccinations^35^.

#### Birth order

The other aspect that determined the child’s incomplete immunization status was their birth order^24^. As birth order increased, the chance of completing a child’s vaccinations decreased^23^. There was a significant correlation between incomplete immunization and being the second or later-born child in the family^25^.

#### Child size at birth

The size of the child at birth is thought to be a factor in incomplete immunization because mothers of average-sized children are more likely than mothers of large-sized children to visit child health care services, such as immunization programs, to keep their child healthy^24^^,33^.

#### Preceding birth interval

The likelihood of a mother fully immunizing her child was lower in those who gave birth before 24 months of the previous delivery than in those who gave birth at 24 months or later^10^. Given that shorter childbearing intervals have been associated with more severe financial, emotional, and psychological impacts, mothers may decide not to use children’s immunization programs^36^. As of right now, studies have been distinguished by the prediction of immunization in children under the age of five, and related to current machine learning approaches, to be reviewed as listed below.

In the study^37^, have predicted childhood vaccination among children aged 12–23 months in Ethiopia with 95.53% accuracy by applying machine learning models using RF, AdaBoost, KNN, SVM, DT, PART, J48 classifier, and Multilayer perception. So, the researchers conducted childhood vaccination among children only aged 12–23 months based on the predictors. Furthermore, Important variables selected based on a best-performance PART model were used to predict childhood vaccination with better generalization and high accuracy.

In the study^38^, proposed a Routine Immunization Program for children at high risk using predictive analytics like Recursive partitioning, SVM, RF, and C-forest models, with an accuracy result of 78.9% achieved Recursive partitioning model. For instance, the researchers were allowed to identify high-risk children corresponding to the default from a Routine Immunization Program. To alleviate the risk factors of children through immunization, as a result, the researchers were not allocated the ensemble learning algorithms.

^39^ researchers proposed the prediction of under-five mortality in Ethiopia using Machine learning approaches. However, the study used ML models like RF, KNN, and LR, with an RF model performance accuracy result of 67.2%. also described the predicted under-five mortality risk factors in Ethiopia. However, the model performance shows relatively low accuracy, and the study doesn’t prove that the ensemble model enhances the model performance and alleviates the fitting of the processing data.

Additionally, the study by^40^ have explored the prediction of incomplete immunization among under-five children across East Africa using machine-learning approaches fitted through LR, RF, KNN, ANN, SVM, NB, XGBoost, and DT. Though the study was intended for children under five years of age, the focus was controversially narrowed to the age group of 12–35 months, and despite utilizing a large dataset of 27,691 cases, the study encountered relatively low model performance, with the highest XGBoost model result an accuracy of 79.01%. but the study also helps to identify the factors that contribute the incomplete immunizations across each specific country with the association rule of mining techniques.

## Methodology

### Study design and settings

**Fig. 1:**
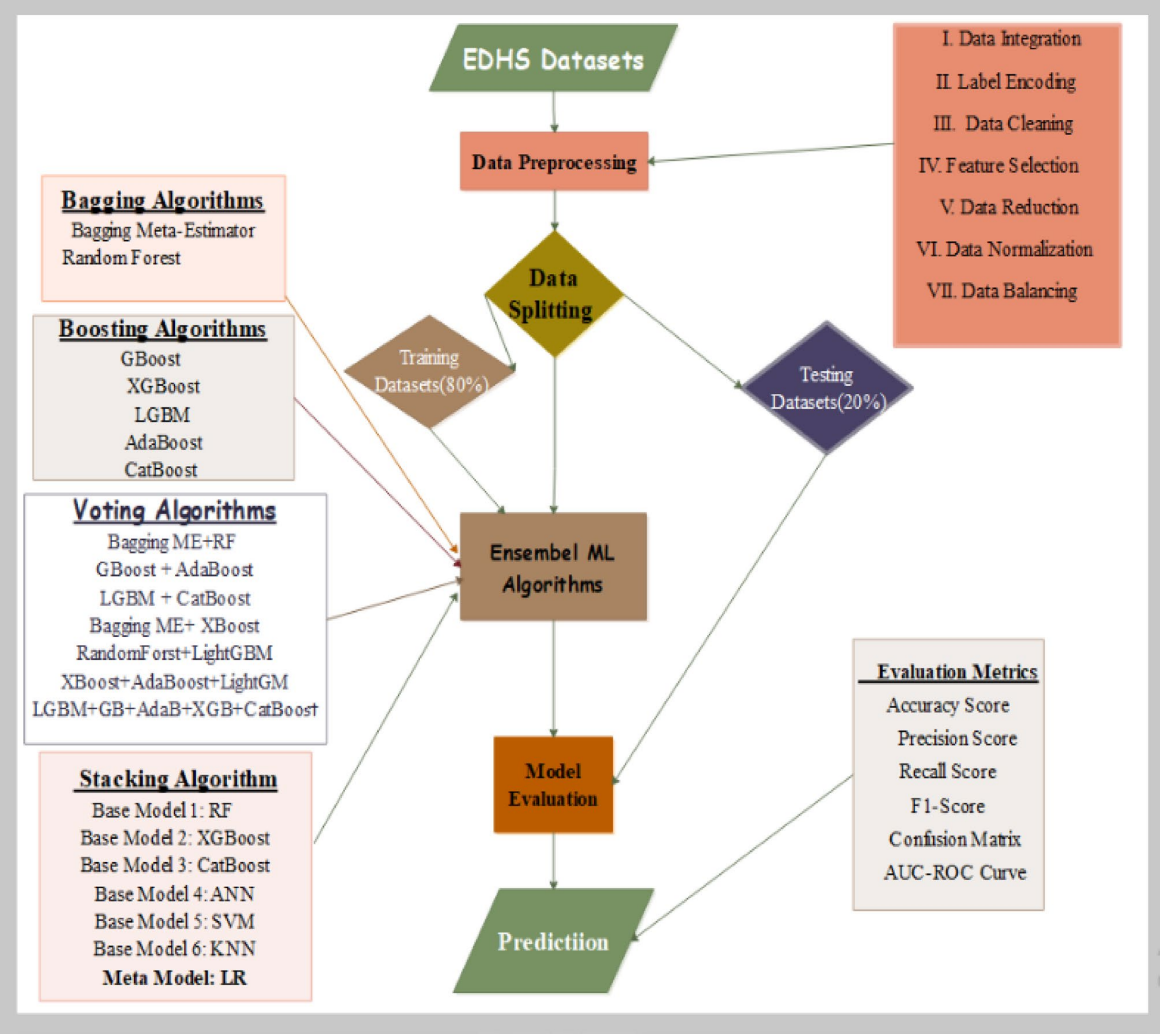
Proposed Study Workflow.

To illustrate the procedural flow of the proposed workflow employed through machine learning methodologies, such as data collection, data preprocessing, data splitting, model development, model evaluations, and others, in this study, the sequential steps were undertaken to enhance the prediction performance of incomplete immunization using an ensemble machine learning technique. The under-five-age children dataset, obtained from the Ethiopian Demographic and Health Survey (EDHS*)* 2016 and 2019 community was utilized for this study. In the data, preprocessing techniques were applied data integration, data labeling, data cleaning, normalization, and balancing methods (class weight, SMOTE, and Tomek link). Also, we utilized data splitting 80:20 ratio for the training and testing data, respectively. After the data splitting, we can build each ensemble model (bagging, boosting, voting, and stacking) algorithm. Finally, to measure the performance of the model by different evaluation metrics. Initially, the necessary Python library packages were installed in Jupyter Notebook. EDA also assists us in selecting which feature selection to consider when building our EML model^41^. The research design was used by applying an algorithm and objectively assessing its effectiveness and efficiency that solving the given problems^42^. For instance, part of conducting the research was merging EDHS 2016 and Ethiopian Mini Demographic and Health Survey (EMDHS) 2019 datasets, performing preprocessing, implementing (building the model), and assessing model performance through a methodological framework based on design science research by applying a structured methodology from the design science paradigm to develop and evaluate new machine learning models^43^. Also, it was carried out before the bagging, boosting, voting, and stacking ensemble methods under consideration, which can solve the class imbalance problem through the Synthetic Minority Over-sampling Technique (SMOTE) and Tomek link methods. The following figure shows the workflow of the study (Fig. 1).

### Data preprocessing

Mostly, the healthcare dataset contains missing values and impurities that can affect the effectiveness of the data. To improve the quality and effectiveness of data obtained through data processing^44,]^^45^. So, ensemble learning techniques are used effectively to preprocess the dataset^46^.

### Data integration

The data used in this study were accessed in the Demographic and Health Survey available at http://www.dhsprogram.com. To access the data, someone needs to follow the steps and protocol outlined under the methods section and get the data from the EDHS under the age of five in Ethiopia. In this study, the unit of analysis has a total sample size of 10,641 from the 2016 EDHS^47^. The survey was implemented by the Ethiopian Public Health Institute (EPHI) in collaboration with the Central Statistical Agency (CSA). The actual data collection period was conducted from January 18, 2016, to June 27, 2016, and 5,753 datasets accessed from the Ethiopia Mini Demographic and Health Survey (2019 EMDHS) was implemented by the Ethiopian Public Health Institute (EPHI), in partnership with the Central Statistical Agency (CSA) and the Federal Ministry of Health (FMOH), under the overall guidance of the Technical Working Group (TWG). Data collection lasted from March to June 2019^48^. To facilitate the analysis of learning algorithms, we used a total of 16,394 datasets that were extracted and imported into a Jupiter notebook.

### Inclusion criteria

From the 2016 and 2019 Ethiopian Demographic and Health Survey (EDHS and EMDHS) datasets, we included all children aged 0–59 months (under five years) with complete immunization records and available socio-demographic and maternal characteristics.

### Exclusion criteria

Records that had missing, incomplete, or inconsistent immunization status were excluded from the analysis. Additionally, any records lacking essential predictor variables required for model development were omitted, and duplicate entries identified during the data cleaning process were also removed to ensure data quality.

### Label encoding

It is a technique used to convert categorical data into numerical values. This is often a necessary step in preparing data for machine learning models, which typically require numerical input, and reduces the data to a simple integer format, which can be efficient for certain models.

### Data cleaning

It is applicable for missing values. This removes all the zero values as they have zero worth, which is not possible. Therefore, the value is eliminated. In EDHS 2016 and EMDHS 2019, datasets often suffer from missing values due to various reasons for incomplete immunization records of the children. However, the data pre-processing phase involves handling missing values by mean Imputation^46^.

### Feature selection

Feature selection methods are intended to reduce the number of input variables to those that are believed to be most useful to a model and to predict the target variable^46^.

#### Dependent variable

Children’s immunization coverage was a binary outcome that was categorized as 0 = no (children who had not completed the full dose of vaccination)^5^. It refers to children in the age group of 0–59 months who missed one or more doses of the recommended vaccine based on the EPI program^23,]^^28^ and 1 = yes (those who had taken the full dose of recommended vaccination) for the classification analysis^49^.

#### Independent variables

The determinant factors for child immunization were extracted from EDHS 2016 and EMDHS 2019. From previous research, baseline explanatory variables were selected^6,8,9,12,28,50,51^. Educational level, Mother’s age, marital status, mother’s and husband’s occupation, Knowledge of Immunization, place of residence, media exposure, wealth index, place of delivery, religion, ANC follow-up, and PNC visit, sex of the child, age of the child, birth order, child size at birth, and preceding birth interval were used as an independent variable or features.

### Operational definition

#### Incomplete immunization

“Children who began vaccination and missed at least one dose of the recommended vaccinations during the period of 0 to 59 months or 0 to 5 years” ^9,^^23^.

#### Complete immunization

“When children had been vaccinated for all recommended vaccinations (one dose of BCG, three doses of polio, three doses of DPT, one dose of measles, three doses of pentavalent, three doses of PCV, and two doses of rotavirus)” ^12,^^28^.

### Data reduction

It uses multiple techniques, such as the feature selection method, to remove noisy data, eradicate redundant data, and transform it into efficient, presentable data.

### Data normalization

Normalize or standardize the features to ensure they are on a common scale. We used the standard feature scaling method that ensures all features contribute equally to the learning process.

### Data balancing

The data transformation can be used to convert data into a format that aids in building efficient machine-learning models and deriving better performance^46^. Though we used some addressing class imbalance techniques through SMOTE Tomek combines both oversampling (using SMOTE for the minority class), under-sampling (using Tomek links to remove Tomek pairs), and class weights can help to ensure that the model pays more attention to the minority class, improving its performance on less frequent classes^15^.

### Data splitting

Data splitting is an important process in machine learning. This involves separating the dataset into distinct subsets: the training set and the test set. we used the dataset, splitting 80% for the training data and 20% for the testing data. After the data partition, we applied data balancing exclusively to the training data to balance the class distribution using SMOTE with Tomek links, leaving the test data unchanged.

### Data validation

It is used to describe the process of checking the accuracy and quality of source data to ensure accurate output and to determine whether the model can correctly identify new data or if it is overfitting the original dataset. Though for predicting incomplete immunization validation, the data were used K-fold cross-validation technique with different evaluation performance predictive models^52^.

#### Programming language

Various programming languages can be used to implement prediction systems. However, we used the Python programming language for this purpose. Python is particularly preferred due to its simplicity and its extensive support for ensemble machine learning algorithms, and can also be easily loaded into different libraries that can perform their tasks and preprocess the dataset.

### Ensemble machine learning algorithms

Ensemble learning is a supervised machine-learning technique that enhances accuracy and perseverance in forecasting by merging predictions from multiple models^53^. The motivation for using ensemble models is to reduce the generalization error of the prediction. As long as the base models are diverse and independent, the prediction error decreases when the ensemble approaches are used^16^. Therefore, the most widely used ensemble machine-learning approaches that we applied in this work are listed below.

#### Bagging ensemble learning algorithms

This algorithm is an ensemble approach that tries to resolve overfitting for classification problems and is also known as Bootstrap aggregating. It does this by taking random subsets of an original dataset, with the substitute, and fitting a classifier (for classification) to each subset^54^. In this study, the popular Bagging algorithms were used as shown below: -.

#### Bagging meta-estimator algorithm

It is an algorithm that can be utilized for predictions in classification through Bagging Classifiers, which are available in the Scikit learning library^55^. Also, this algorithm is particularly effective in reducing overfitting, which is a common problem in machine learning models.

### Random forest algorithm

Random Forest is a versatile ensemble learning algorithm that combines the power of multiple decision trees to make robust predictions, and it works based on the concept of ensemble learning, which is a process of combining multiple classifiers to solve a complex problem and to improve the performance of the model^56^.

### Boosting ensemble learning algorithms

Ensemble learning has been utilized in several real-life problems. For instance, in healthcare, ensemble learning has gained significant popularity due to its effectiveness in predicting, detecting, diagnosing, and prognosing various diseases. Even though boosting algorithms are getting more and more popular, and powerful for reducing underfitting^57,]^^58^^,59^. we performed the following five boosting algorithms as ensemble learning approaches: -.

### Gradient boosting algorithm

The GB method sequentially trains weak learners, with each estimator being added one by one by adjusting their weights^57^. The main goal of this algorithm is to predict residual errors from earlier estimators and reduce the difference between anticipated and actual values, which improves the overall predictive performance.

## Extreme gradient boosting algorithm

XGBoost operates by integrating diverse types of decision trees, also known as weak learners, to independently compute similarity scores by incorporating gradient descent and regularization techniques, to modify the gradient descent and regularization procedure, and aids in overcoming the issue of overfitting during the training phase effectively^57^.

### Light gradient boosting machine algorithm

LightGBM is an extension of a gradient boosting algorithm framework based on a decision tree to increase the efficiency of the model. Although capable of handling large datasets with less memory utilization, the model evaluation process, reducing the number of features in sparse datasets and during training with faster speed and resulting in better accuracy^57^.

### Adaptive boosting algorithm

This algorithm operates by dynamically adjusting weak learners’ weights without prior knowledge. During the training process, the weakness of each base learner is evaluated based on the estimator’s error rate^57,]^^60^. Even though this algorithm improves generalization by alleviating overfitting and improving performance.

#### Categorical boosting algorithm

The CatBoost is faster than other boosting algorithms, as it does not require the exploration of data preprocessing^57^. And also enables efficient CPU implementation, reduces prediction time, facilitates fast model application, and acts as a form of regularization to prevent overfitting.

#### Voting ensemble learning algorithm

Ensemble learning is a powerful technique in machine learning where multiple models are combined to improve overall predictive performance and robustness^61,]^^62^. This method contains one unit for prediction and one unit for the optimization of the prediction unit to reach an accurate output^59^. In this study, we used a voting approach with the role bagging and boosting approaches independently, and each base model for each class and calculates the weighted average of these probabilities to make the final prediction through soft-voting^63^.

### Stacking ensemble learning algorithm

This algorithm is the most popular ensemble learning strategy in machine learning that combines the predictions of numerous base models to get a final prediction with better performance^59^. It enables us to train multiple models to solve similar problems, and based on their combined output, builds a new model with improved performance and avoids overfitting^64,]^^65^.

**Fig. 2:**
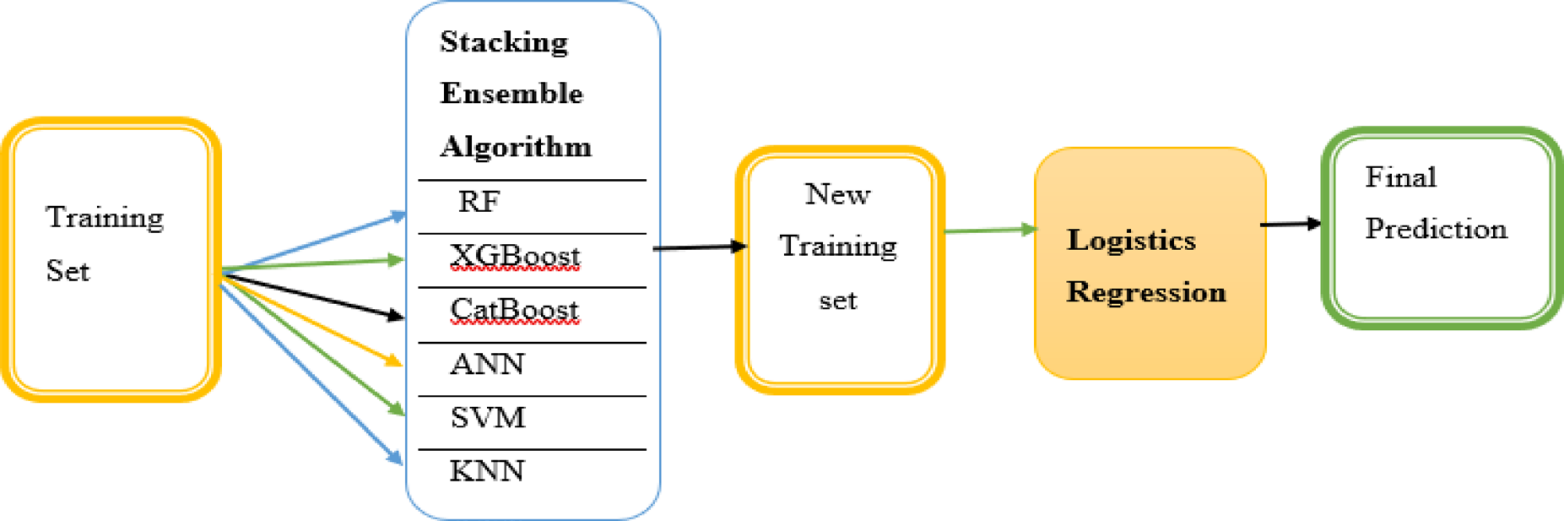
Stacking Workflow.

The primary idea of stacking was to feed the predictions of numerous base models into a higher-level model known as the meta-model or blender, which combines them to get the final prediction of incomplete immunization for children^59^. In this study, the stacked ensemble model used Random Forest, XGBoost, CatBoost, ANN, SVM, and KNN classifiers as the base models and logistic regression as a meta-model (Fig. 2), which predicts the output using the data and the predictions from the base models^66^.

### Random forest algorithm

Random forest, or random decision forest, is one of the popular supervised machine learning techniques for classification, regression, and other tasks that operate by constructing a multitude of decision trees at training time. Also, it is built on the notion of ensemble learning, which is a method that involves integrating several classifiers to solve a complicated issue and enhance the model’s performance.

### XGBoost algorithm

This algorithm is an advanced implementation of the gradient boosting algorithm and has proved to be a highly effective algorithm, extensively used in machine learning competitions and also. It also has high predictive power, and it consists of a variety of regularizations that reduce overfitting and improve overall performance.

#### CatBoost algorithm

This algorithm is used to handle a categorical variable in a tedious process, especially when we have a large number of such variables. Also, the categorical variables have too many labels (i.e., they are highly cardinal), so performing one-hot-encoding on them exponentially increases the dimensionality, and it becomes really difficult to work with the dataset. Besides this, the approaches can automatically deal with categorical variables and do not require extensive data preprocessing.

### Artificial neural networks

This algorithm is the most popular today. An ANN is a non-linear model that is widely used in Machine Learning^67^. Also, it is a computational network in science that resembles the characteristics of a human brain. So, it consists of a large number of interconnected neurons that are inspired by the workings of the brain. These neurons have the capabilities to learn, generalize the training data, and derive results from complicated data. Even though the model consists of weights or synaptic connections, the learning rule is used for adjusting the weights and activation functions of the neuron^68^.

#### Support vector machine algorithm

SVM is one of the most popular supervised learning algorithms, which is used for classification as well as regression problems^69^. However, primarily, it is used for classification problems in machine learning with resilience to overfitting, and it gives us the flexibility to define how much error is acceptable in our model and find an appropriate line (or hyperplane in higher dimensions) to fit the data.

#### K-nearest neighbors algorithm

KNN is one of the simplest forms of supervised machine learning algorithms, mostly used for classification, and it classifies the data point based on how its neighbor is classified. Even though KNN classifies the new data points based on the similarity measure of the earlier stored data points^70^.

#### Logistic regression

LR is a supervised machine learning algorithm that accomplishes binary classification tasks by predicting the probability of an outcome, event, or observation^71^. It has better stability than the other six base models. However, we explored that the LR meta-learning algorithm better adapts to all of the downstream tasks.

#### Evaluation metrics

Evaluation metrics are quantitative measures used to assess the performance and effectiveness of a statistical or machine-learning model. When evaluating a machine learning model, it is crucial to assess its predictive ability, generalization capability, and overall quality commonly used^17^,^73^.

#### Accuracy

Accuracy is a fundamental evaluation metric for assessing the overall performance of a classification model. It is the ratio of the correctly predicted instances to the total instances in the dataset. The formula for calculating accuracy is:

1 $${\rm Accuracy = \frac{TP+TN}{TP+TN+FP+FN}}$$

#### Precision

Precision is the proportion of true positive predictions among all positive predictions. It is a measure of how accurate the positive predictions are.

2 $${\rm Precision = \frac{TP}{TP+FP}}$$

#### Recall

Recall, also known as sensitivity or true positive rate (TPR), is the proportion of true positive predictions among all actual positive instances. It measures the classifier’s ability to identify positive instances correctly.

3 $${\rm Recall =\:\frac{TP}{TP+FN}}$$

Where, TP = True Positive TN = True Negative FP = False Positive FN = False Negative.

#### F1-Score

The F1-score is the harmonic mean of precision and recall, providing a metric that balances both measures. It is beneficial when dealing with imbalanced datasets, and is expressed as:

4 $${\rm F1\_Score =\frac{2}{\frac{1}{Precision}+{\frac{1}{Recall}}}} \:=\: {\rm \frac{2\: X\: Precision\: X\: Recall}{Precision + Recall}}$$

#### Confusion matrix

A confusion matrix, also known as an error matrix, is a tool used to evaluate the performance of classification models in machine learning and statistics^73^. It presents a summary of the predictions made by a classifier compared to the actual class labels and provides a comprehensive view of the model’s performance, including each class’s correct and incorrect predictions.

#### AUC-ROC curve

The AUC-ROC curve, or Area Under the Receiver Operating Characteristic curve, is a graphical representation of the performance of a binary classification model at various classification thresholds^17^,^75^. ROC stands for Receiver Operating Characteristic, and the ROC curve is the graphical representation of the effectiveness of the binary classification model. It plots the true positive rate (TPR) vs. the false positive rate (FPR) at different classification thresholds.

### Organizations of the study

The Ethiopian Demographic and Health Survey (EDHS) is a national survey conducted to collect data on various health indicators across Ethiopia. It is part of a larger Demographic and Health Surveys (DHS) Program, conducted globally in collaboration with various countries’ health ministries and statistical agencies. The Ethiopian Demographic and Health Surveys (EDHS), including both the 2016 EDHS and the 2019 Ethiopia Mini Demographic and Health Survey (EMDHS), were comprehensive national surveys conducted to gather key data on health and demographic indicators. These surveys provide valuable insights into areas such as maternal and child health, family planning, immunization coverage, nutrition, HIV/AIDS, and mortality rates across Ethiopia.

The 2016 EDHS was conducted by the Central Statistical Agency (CSA) of Ethiopia, in collaboration with the Ministry of Health (MoH). This survey collected detailed data on various health-related factors, including child immunization coverage. The 2016 EDHS is widely cited and forms a basis for understanding trends in health services, including maternal and child healthcare, across Ethiopia. In 2019, EMDHS was a smaller, follow-up survey to the 2016 EDHS. The 2019 Mini Demographic and Health Survey (EMDHS) focuses on specific indicators to provide updates between the full-scale surveys. It was implemented by the Ethiopian Public Health Institute (EPHI) in collaboration with the MoH, CSA, and technical support from ICF International. This survey provides updated data on child immunization, maternal health, and key health service indicators, helping to monitor progress made since 2016. So far, the 2016 EDHS and 2019 EMDHS datasets are crucial for this research, as they offer comprehensive data on the immunization status of children aged 0–59 months in Ethiopia.

### Experimental setup

All of the models compiled for this study were run in a Jupyter Anaconda environment. We used a DELL laptop running Windows 10 with 32-bit and powered by an Intel (R) CPU with 1.20 GHz, and 8 GB of RAM, and the dataset was extracted by using Stata 14.2 software. As far as all experimental studies were carried out in the Scikit-learn and TensorFlow framework, a free and open-source machine learning toolkit created in Python 3.9 that executes on Jupyter Notebook, Brave web browser, for accessing of downloading, Edraw Max 9.4 for drawing purposes, and additionally, used GitHub code spaces to push all codes.

## Results

### Machine learning analysis of children’s immunization

This study focused on immunization among children under five in Ethiopia. We have to investigate the children’s age and compare it to the basic recommended vaccination from the Expanded Program on Immunization (EPI) in Ethiopia.

### Age distributions of the study

Based on Exploratory Data Analysis (EDA), which is used to visualize the relationship between age groups and vaccination status to see if certain age groups are under-vaccinated, and help to analyze the percentage of children in each age group who received each vaccine^41^.

**Fig. 3:**
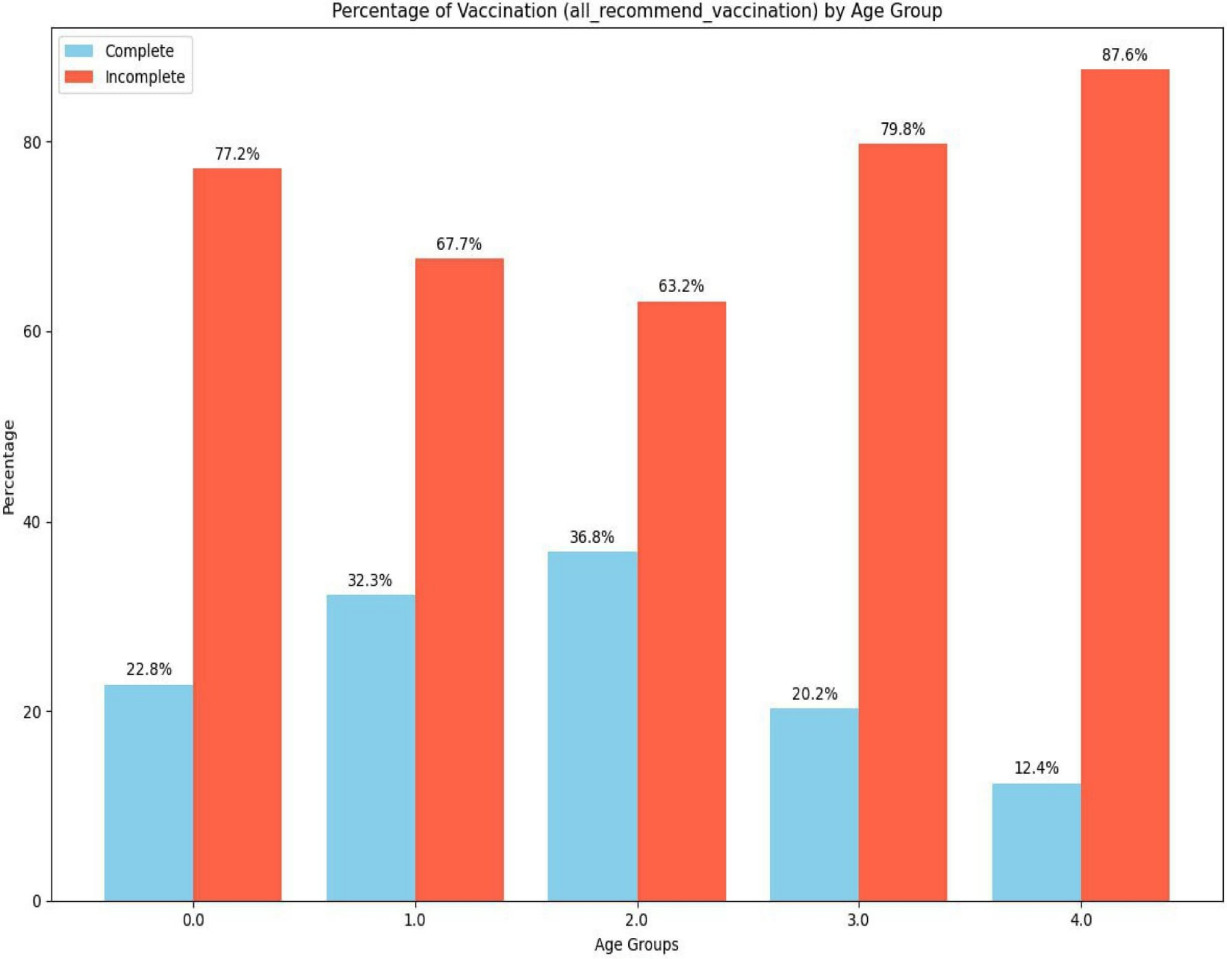
Recommended vaccination coverage with each age group.

This study illustrates the above dataset distribution of the percentage of vaccination completion (all recommended vaccinations) across different age groups. The figure compares the percentage of children with complete and incomplete vaccinations within each age group (Fig. 3).

In all age groups, incomplete immunization is predominant. The percentage of children with incomplete immunization is significantly higher than those with complete immunization. However, the trend suggests that as children grow older, the likelihood of having incomplete immunization increases, with the highest incomplete immunization rate observed in the 4-year-old age group. While the percentage of complete immunizations increases slightly between the 0, 1, and 2 age groups, it declines again in the older age groups (3 and 4).

Therefore, this chart highlights the critical challenge of ensuring complete immunization among children and suggests that targeted interventions may be needed, especially as children grow older, to ensure they receive all recommended vaccinations.

### Recommend vaccination coverage from EPI

**Fig. 4:**
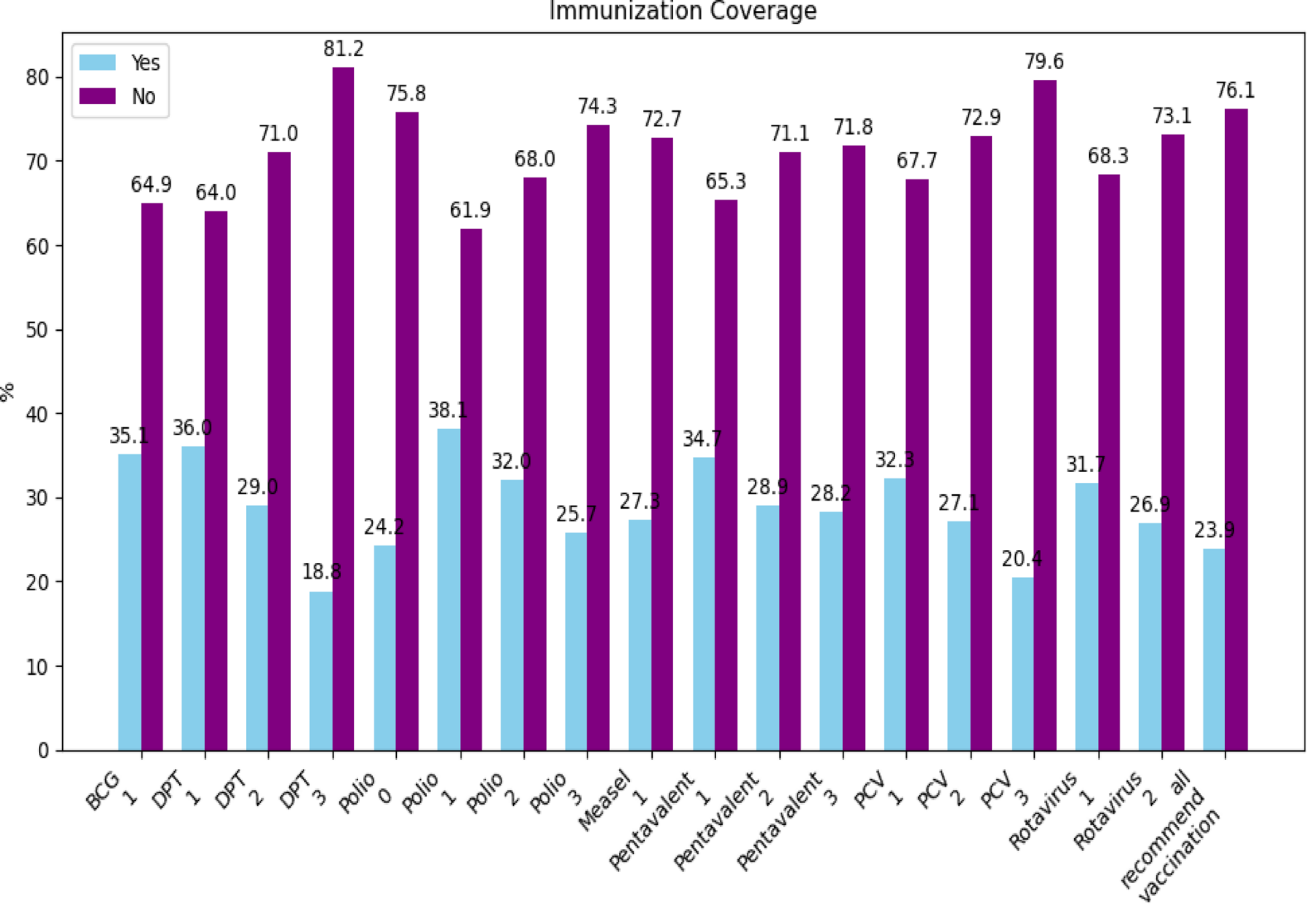
Immunization Coverage Status among the under-five age.

The proposed study, which aims to predict incomplete immunization among children under the age of five in Ethiopia using ensemble learning algorithms and determine the factors behind incomplete immunized, addresses several key gaps in current public health efforts and research., The study’s findings will help policymakers better understand which vaccines are underutilized and which demographic or social factors contribute to incomplete immunization (Fig. 4).

According to the above immunization coverage status, incomplete (no) immunization status needs more focused interventions than the complete (yes) immunization status. However, this study applied ensemble learning models such as bagging, boosting, voting, and stacking to predict incomplete immunization in Ethiopian children. As a result, this innovative approach improves upon traditional approaches, which may not effectively capture the complex interactions between incomplete immunization factors.

### Balanced sampling techniques for addressing class imbalance

**Fig. 5:**
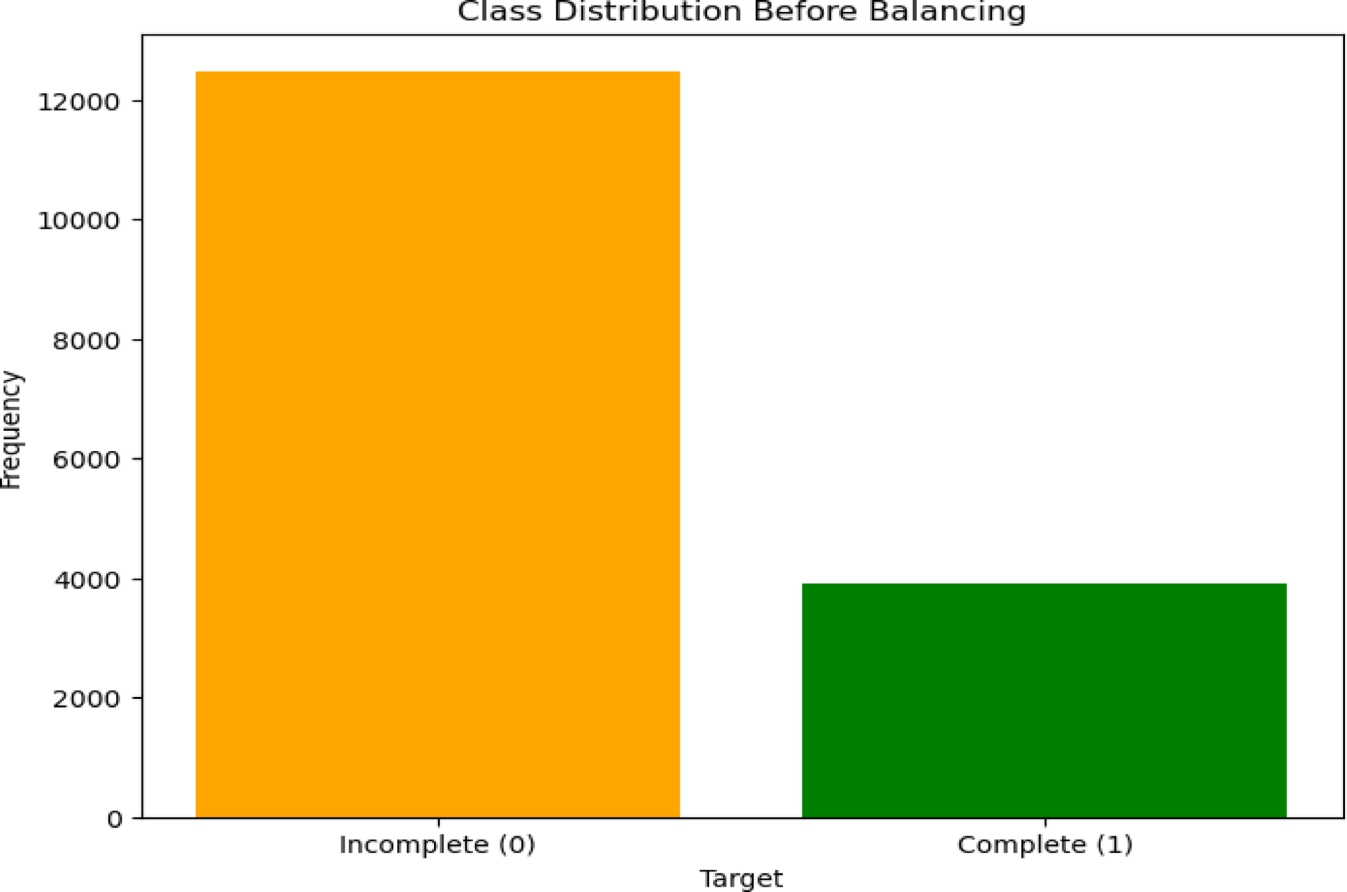
Class Distribution before Balancing.

Unbalanced data handling was a key strategy for this study, so we need to handle the problem of unbalanced data and boost the performance of the ensemble machine learning algorithms. From the outcome feature 12,483 of the observations were classified as incomplete, and 3,911 were complete on the original data (Fig. 5).

**Fig. 6:**
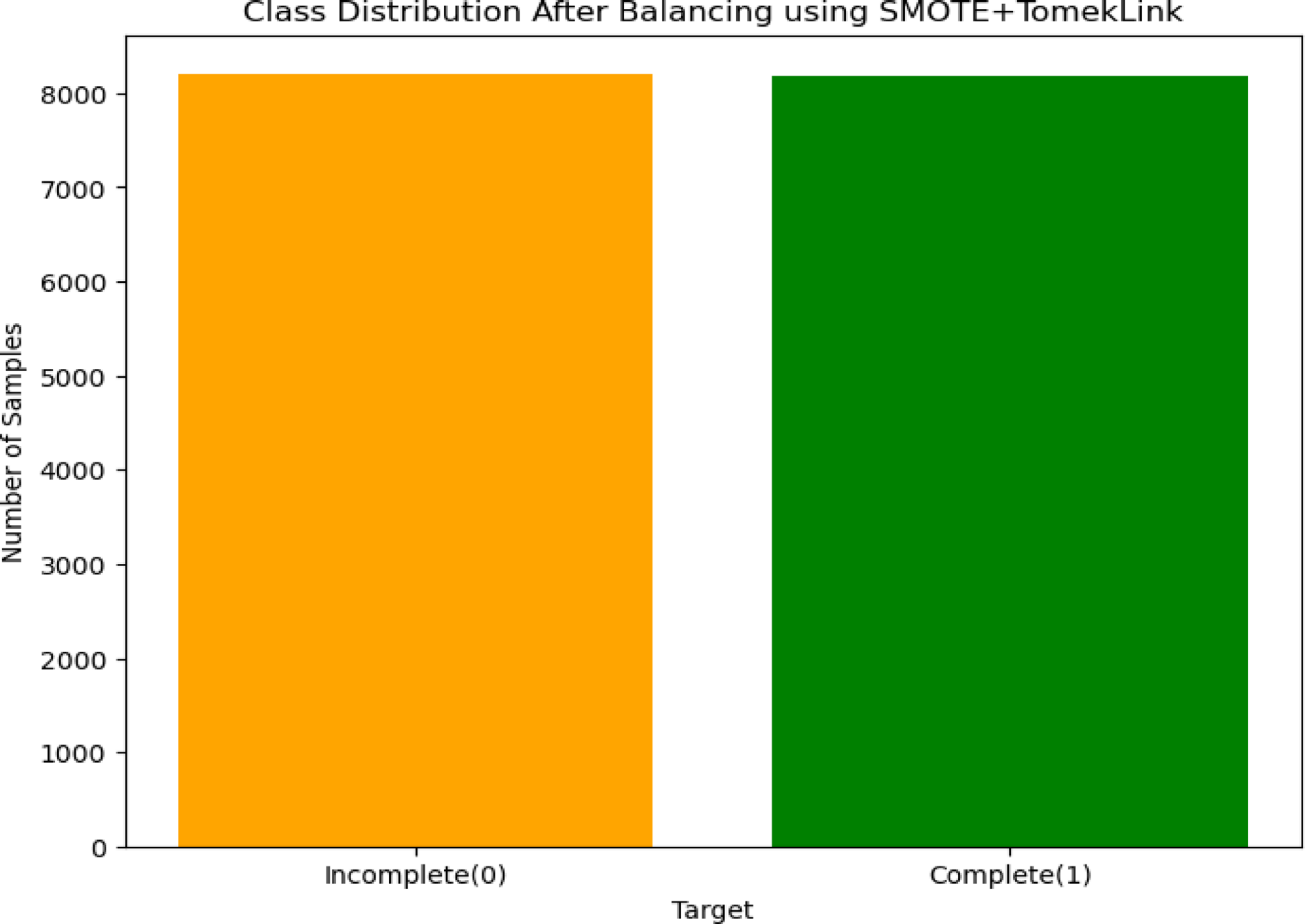
Class Distribution After balancing using SMOTE and Tomek Link.

The balanced sampling method we used was a different sampling technique to balance the unequal dataset by either decreasing the majority class or maximizing the minority class weights using a specific weight of [0.5154, 0.897]^75^. By assigning these weights, the model gives more importance to the minority class during training to avoid bias toward the majority class. combining this with techniques of SMOTE and Tomek Links for resampling can help address class imbalance effectively^15^. So, after applying the balanced method, the training data with the majority class datasets are 6,583, and the minority class is also 6,532 datasets. The proven model was fine-tuned and reflects improved performance results (Fig. 6).

### Ensemble models evaluation results

Model Evaluations were performed for all the training and testing results, where we evaluated our model using the evaluation measurement matrices are accuracy, precision, recall, and F1-score^17,]^^44^ and we allocate the overall prediction of the model as decided by accuracy.

### Training evaluation results

**Fig. 7:**
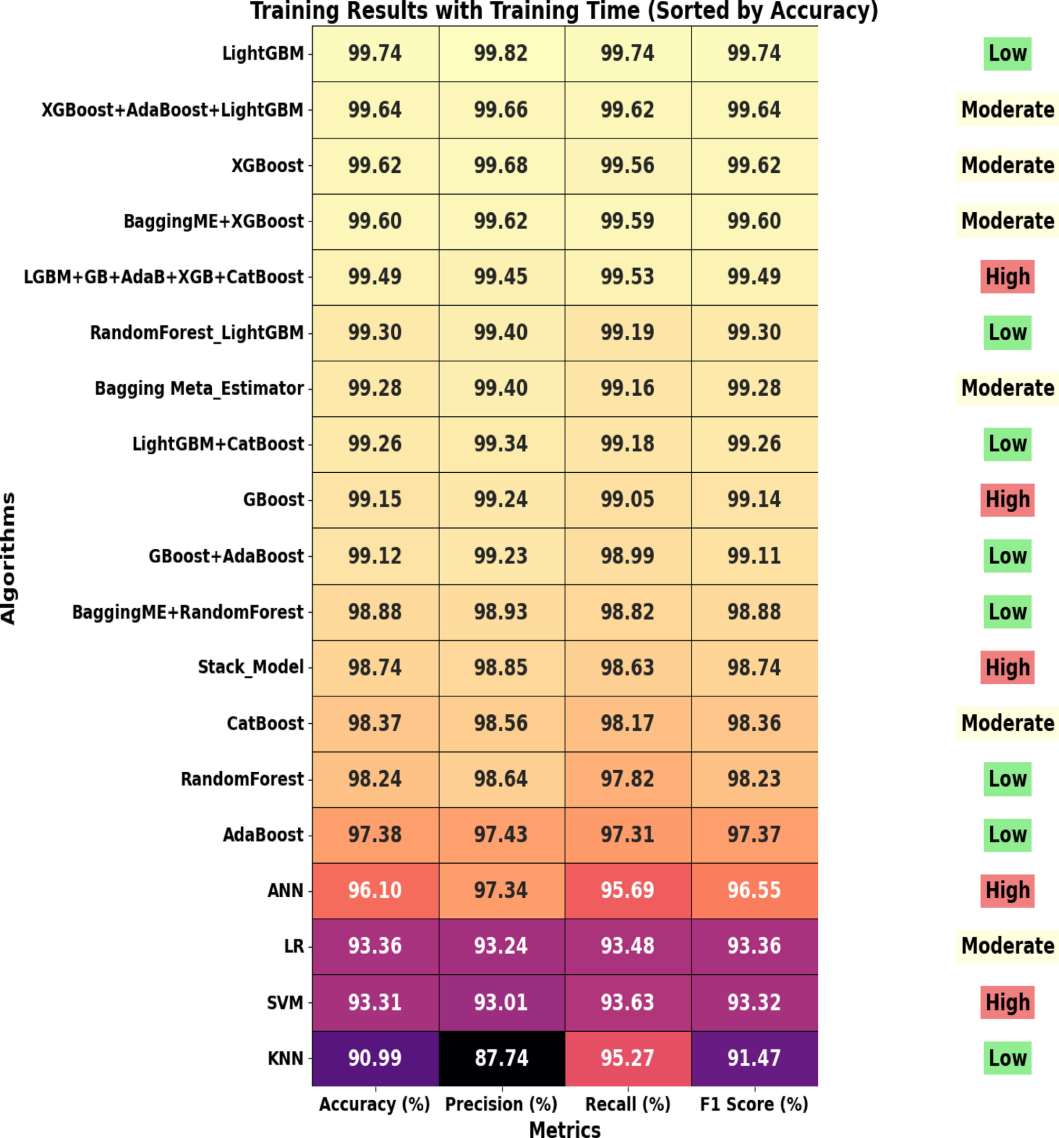
Training Set Results.

During the training phase of the study, various ensemble machine learning models were evaluated on their ability to predict incomplete immunization in children using the balanced dataset (Fig. 7).

### Cross-Validation accuracy result

K-fold cross-validation is a technique used to evaluate the performance of a machine learning model by splitting the dataset into multiple subsets (folds) and assessing the model’s performance through various iterations.

**Fig. 8:**
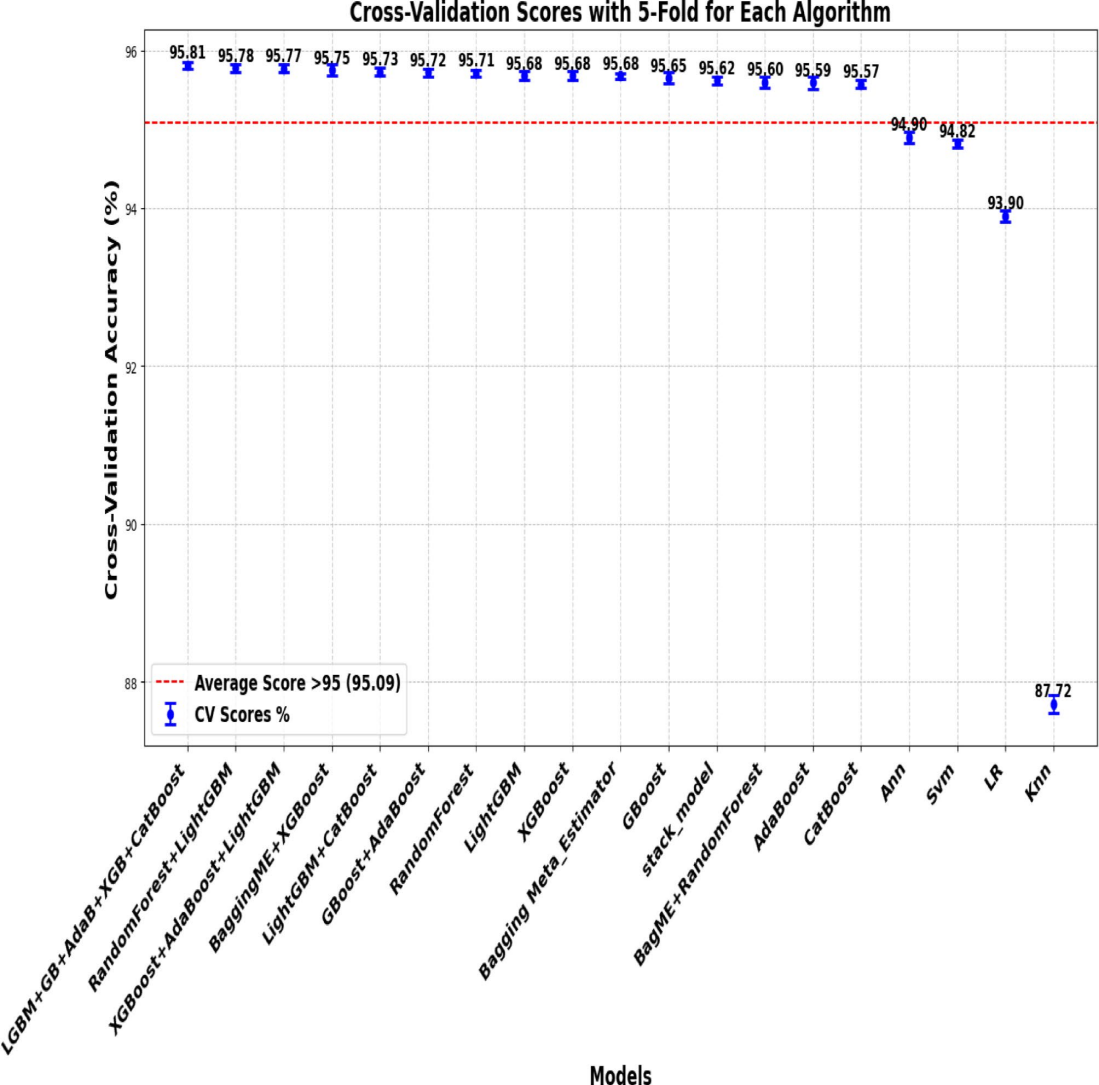
Cross Validation Scores.

In this study, we used 5-fold cross-validation as a practical approach to assess the performance of a machine learning model, providing insights into how well the model generalizes to unseen data. It offers a robust measure of model performance by averaging the results from multiple train-test splits (Fig. 8).

### Testing evaluation results

**Fig. 9:**
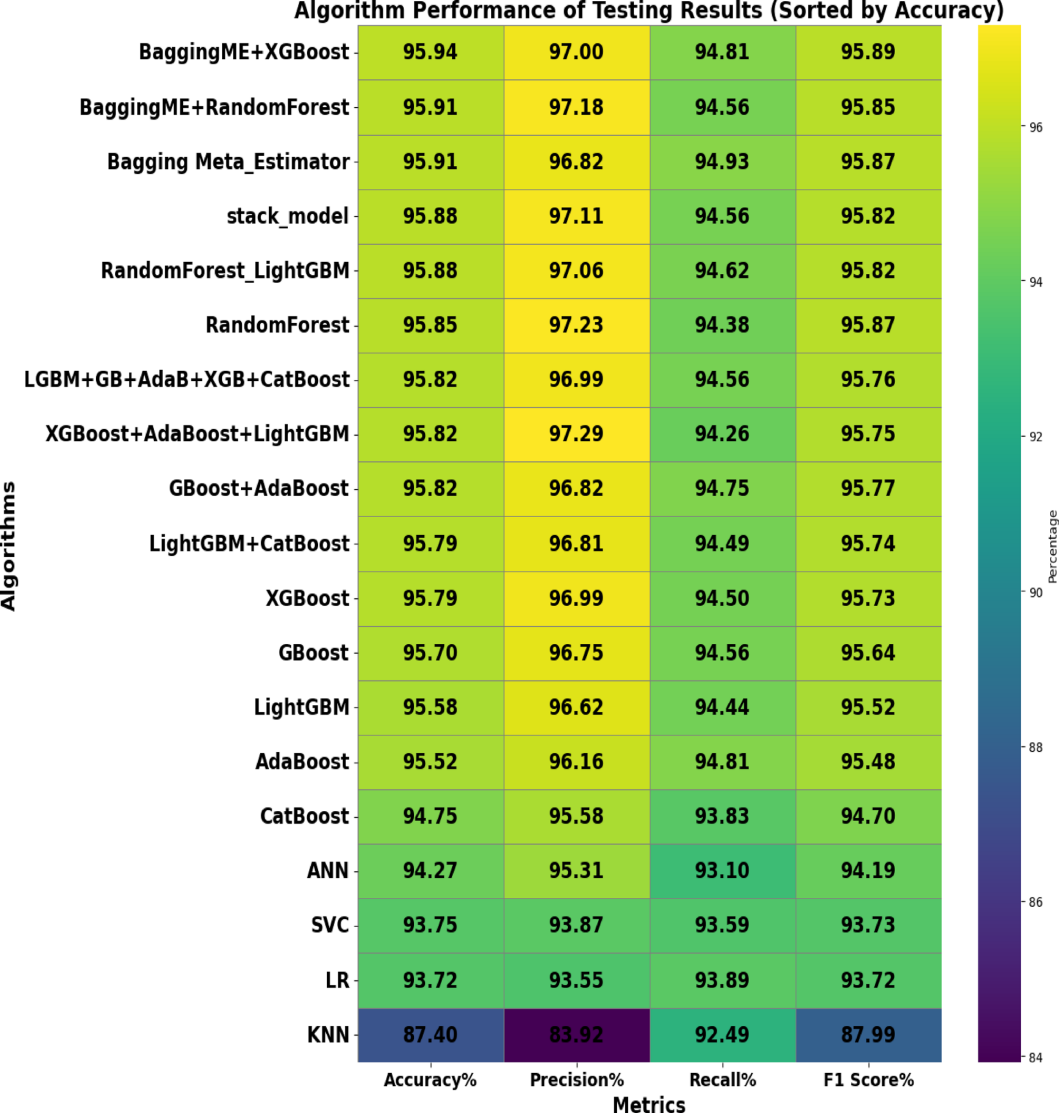
Testing Set Results.

The machine learning models were used to evaluate the results of testing data with different evaluation metrics, ensuring accurate measuring of the models through accuracy, precision, recall, and f1_score of the testing data for each model performance that we have evaluated (Fig. 9).

The testing result allows for a direct comparison of the predictive performance of the different ensemble techniques. Based on the performance accuracy, the Bagging Meta-Estimator with XGBoost voting ensemble model appears to be the best-performing ensemble model for predicting incomplete immunization factors analysis in this study. These comparative results, along with any additional performance metrics like accuracy, precision, recall, and F1-score, would be an important way to showcase the key findings from the evaluation of the different ensemble algorithms in this study. Bagging ME + XGBoost stands out as the top performer with an impressive accuracy of 95.94%. This model excels in precision (97.00%) and maintains a high recall (94.81%), leading to a remarkable F1-score of 95.89%. So, the combination of Bagging with XGBoost proves more effective, accurate, and reliable than other algorithms. Highlighting the superiority of the Bagging Meta-Estimator model with XGBoost through accuracy, precision, recall, and F1-score would be a key finding to emphasize in the study.

### Confusion matrix

**Fig. 10:**
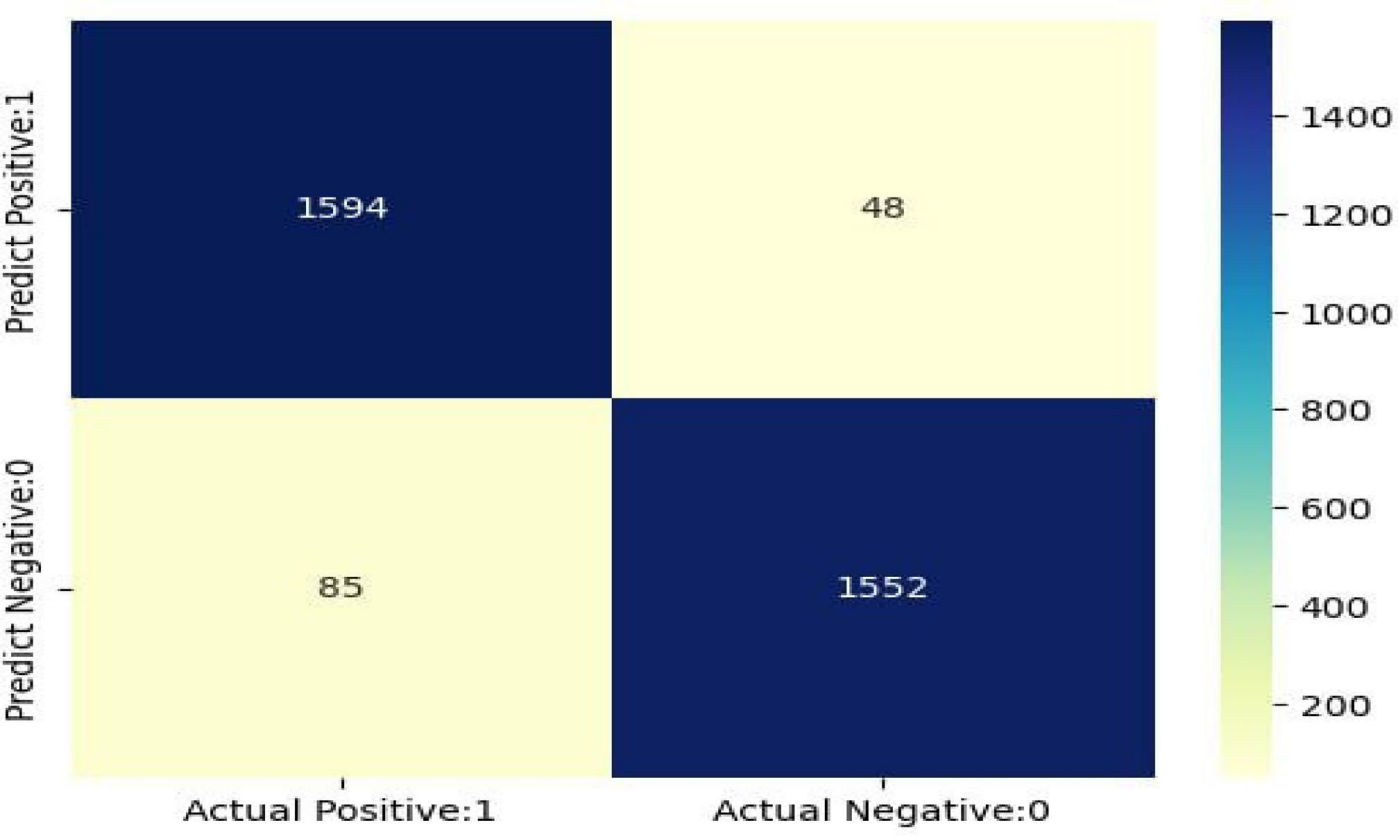
Confusion matrix for Bagging ME+XBoost Algorithm.

In the Meta-Estimator model with the XGBoost ensemble model confusion matrix displays the actual and predicted class values to illustrate how well the model performed. It is an n x n square matrix, with the actual and predicted classes represented in the rows and columns, respectively. As the figure below, the confusion matrix of the Meta-Estimator + XGBoost model proved that the actual and the predicted results of the testing data (Fig. 10).

After training, 3279 test datasets were utilized to assess the Bagging ME + XBoost model performance. From 1642 incomplete immunization statuses, the model correctly predicted 1594 of them as incomplete (true positive). And from 1637 complete, the model predicted 1552 true negatives. However, the model misclassified 48 false positives and 85 false negatives. Overall, the model was predicted with accuracy, precision, recall, and F1-score on test data.

**Accuracy** = TP + TN/(TP + FP + FN + TN) = > 1594 + 1552 ∕ 1594 + 48 + 85 + 1552 ≈ 95.94%.

**Precision** = TP/(TP + FP) = > 1594∕ 1594 + 48 ≈ 97.07%.

**Recall** = TP/(TP + FN) = > 1594∕ 1594 + 85 ≈ 94.94%.

**F1-score** = (2 * Precision * Recall)/(Precision + Recall) => (2*0.9494*0.9707)/(0.9494 + 0.9707) ≈ 95.9%.

### AUC_ROC curve

**Fig. 11:**
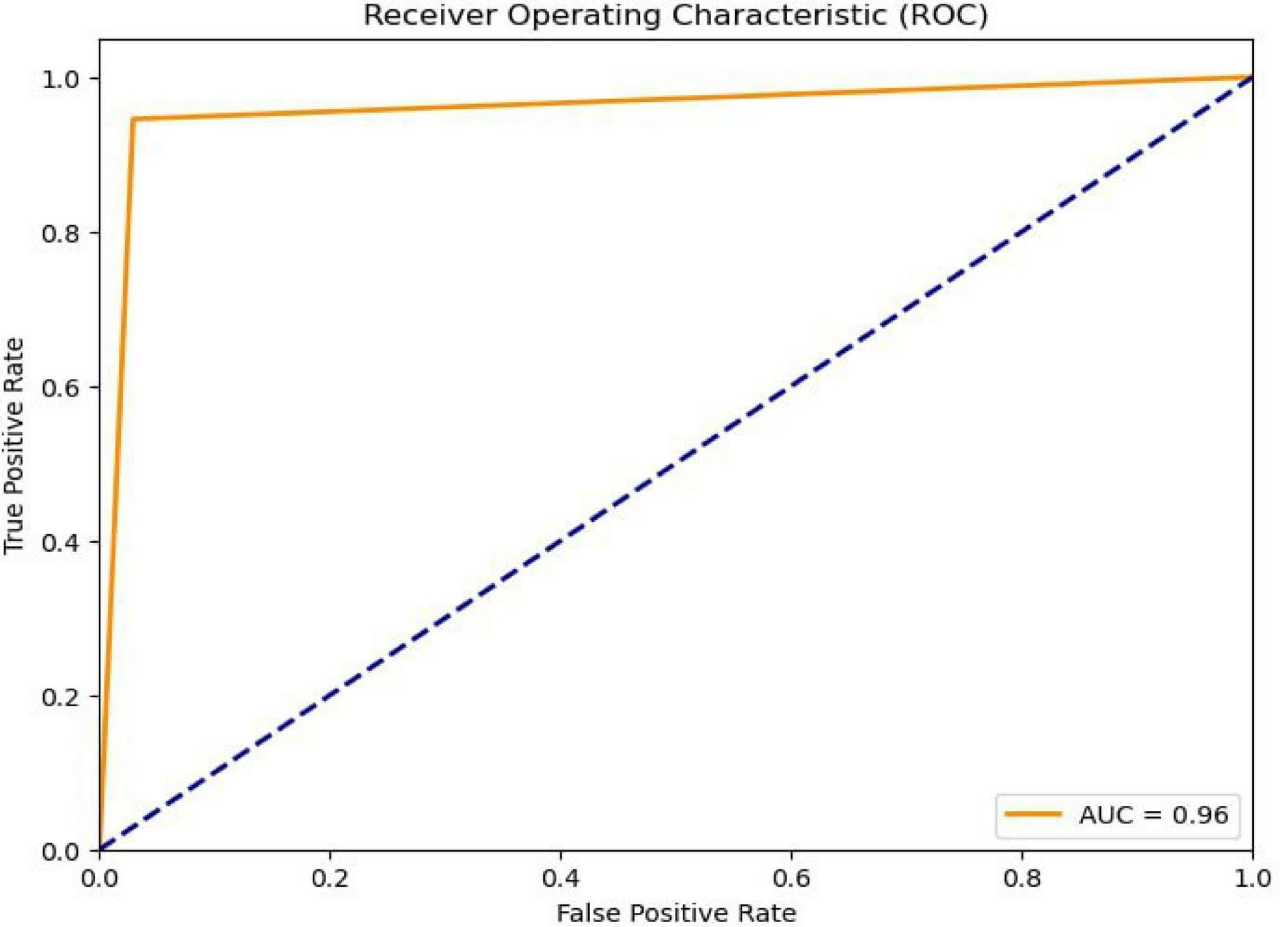
AUC-ROC Curve for Bagging ME+XBoost

The AUC-ROC (Area under the Receiver Operating Characteristic Curve) is a performance metric used to evaluate the quality of binary classification models. The ROC curve plots the true positive rate (TPR) against the false positive rate (FPR) at various threshold settings. The AUC (Area under the Curve) quantifies the overall ability of the model to discriminate between positive and negative classes (Fig. 11).

### Factor analysis of the incomplete immunization prediction model

Using the Bagging Meta estimator model with XGBoost through voting ensemble, the feature importance analysis provides valuable insights into the factors influencing incomplete immunization among Ethiopian children and analyzes these models. First, train the model, extract feature importance through Gini and gain importance, select top features, cross-validate, combine feature importance through a weighting scheme, and finally interpret the results.

**Fig. 12:**
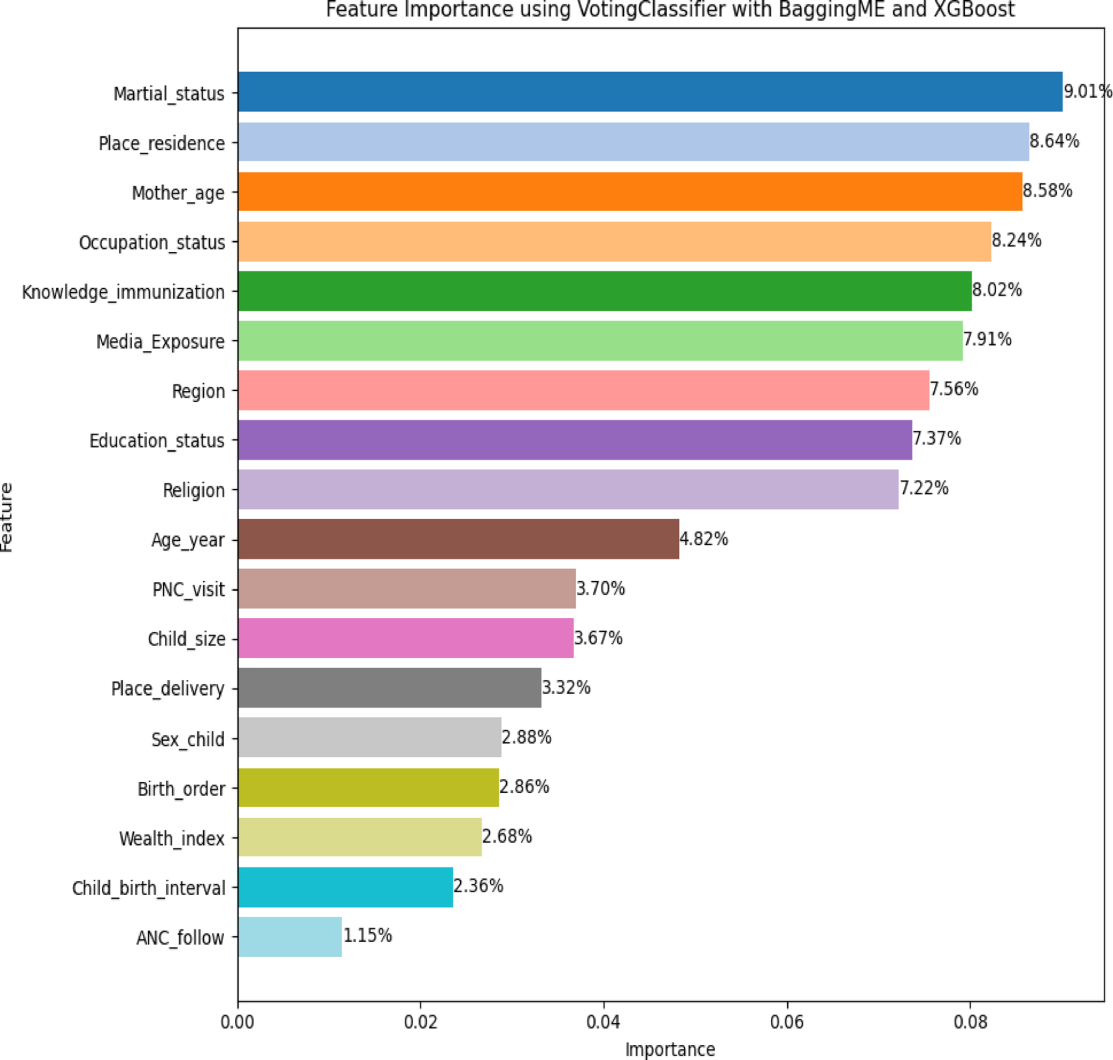
Factors for incomplete immunization using Bagging ME+XGBoost

From the above figure, the model identifies several key factors that significantly impact the likelihood of a child being incompletely immunized (Fig. 12).

## Discussions

### Key factors contributing to incomplete immunization

The analysis highlighted several significant factors that were determined for the best performance results of the Bagging ME and XGBoost ensemble model.

### Factors with the highest influence on incomplete immunization

When analyzing factors influencing incomplete immunization, it’s essential to understand how various socio-demographic factors can affect immunization coverage.

The influence of maritial status (9.01%) suggests the most important factor in the prediction model of the Bagging ME + XGBoost algorithm, indicating that children from single-parent or non-married, divorced households are more likely to be incompletely immunized^24^^,26^^,28^^,29^^,30^^,31^, place of residence (8.64%), employees, the second factor, that living in rural areas or regions with limited healthcare^23^^,26^^,32^. Because infrastructure plays a critical role in incomplete immunization, underscoring the importance of improving healthcare access in these areas, and also the mother’s age (8.58%) is a strong predictor, where younger or older mothers may face barriers to accessing immunization services for their children^22^^,23^^,26^^,27^.

Therefore, the Bagging ME and XGBoost model provides feature-important results and insight into the key factors behind incomplete immunization, which are particularly influential. The findings suggest that maritial status, place of residence, and mother’s characteristics are the primary factors determining whether a child completes their vaccination schedule. Also, the model highlights the importance of knowledge about immunization and media exposure, emphasizing that education status and healthcare outreach programs can have a significant positive impact. Furthermore, factors such as regional healthcare disparities and maternal education status underscore the need for localized interventions that take into account the specific needs of different communities.

This study represents a novel approach to analyzing the factors contributing to incomplete immunizations among Ethiopian children. Throughout the study, an overview of key findings for the four main types of ensemble machine learning algorithms of bagging, boosting, voting, and stacking models. Particularly, voting ensemble models (Bagging meta-estimator with XGBoost) consistently achieve higher accuracy of 95.94%, precision of 97.00%, recall of 94.81%, and F1 scores of 95.89% compared to other approaches with the involvement of hyperparameter tuning. In this case, we used a Grid Search method with five-fold cross-validation that optimizes the hyperparameters of the ensemble machine learning models. For the Bagging meta-estimator, we configured the different parameters like n_estimators = 300 for the number of base estimators (or trees) in the ensemble, max_samples = 0.5 for specifying that each base estimator is trained on a random sample of 50% of the training data. This helps in reducing overfitting, max_features = 0.53 indicates that each base estimator uses 53% of the features, promoting diversity among the base models and enhancing generalization, random_state = 100 sets the seed for the random number generator to ensure reproducibility of results and, bootstrap = True enables bootstrapping, where each base estimator is trained on a bootstrap sample of the data, contributing to the ensemble’s robustness.

In addition, we employed the XGBClassifier parameters such as random_state = 0 to ensure the results are reproducible by initializing the random number generator with a fixed seed, and learning_rate = 0.12 controls the step size at each iteration while moving toward a minimum of the loss function, and was used to balance between convergence speed and model accuracy. Also, we aligned the model’s predictions by tuning the threshold value = 0.52 can control the trade-offs between different performance metrics with soft voting.

This section compares key aspects of the current study with those from previous research, emphasizing the improvements and advancements made.

A study by ^38^, utilized PART algorithms. Reported a maximum accuracy result of around 95.53%, significantly lower than the Bagging meta-estimator + XGBoost in the current study. The gap has small datasets, not enough for preprocessing techniques and ensemble model buildings, focus on the association rule of mining for important attribute selection rather than the predictive results, additionally, adequate ANC visits are the determinant factors for the age of the children 12–23 months using PART model, the current study used a more extensive dataset with 16,394 rows for the age of children 0–59 months and applied advanced ensemble methods like the Bagging Meta Estimator + XGBoost algorithms, achieving significantly higher accuracy (95.94%). Due to these predictive results, marital status and place of residence are the basic determining factors associated with incomplete immunization. The comprehensive model evaluation metrics, such as cross-validation accuracy, precision, recall, and F1-score, were considered to ensure robust model performance.

A study by ^41^, carried out the XGBoost algorithm. Achieved the highest accuracy result of 79.01%. The results were provided with low accuracy. The researchers focus on the age of children between 12 and 35 months with a total dataset of 27,691. but after balancing the total datasets used 20,459 with SMOTE techniques. So, the preprocessing techniques are faced with underfitting and overfitting. However, the current study filled the gap with the best data balancing techniques like SMOTE, Tomek Link, and class weights. The researchers address the number of living children as the top factor of incomplete immunization, but it does not utilize the feature importance with the highest model results of the XGBoost algorithm. However, ensuring a more comprehensive analysis of factors influencing incomplete immunization. As a result, the marital status has the highest score features importance through the Bagging meta-estimator + XGBoost algorithm, reflecting a more accurate and reliable prediction model.

In a study by ^39^, a method that used recursive partitioning and reported an accuracy of 78.9% with low accuracy, not enough for use in preprocessing techniques and it doesn’t prove the ensemble models, The current study’s use of ensemble machine learning models resulted in significantly higher accuracy (95.94%), and those models ensured a more generalizable and accurate prediction results.

In a study by ^77^, Employed XGBoost Approaches, and an accuracy result of 62%.The researchers’ results showed low accuracy, single model use or model limitations, not proving the specific age of vaccination uptake, small data sets were used, and it does not investigate the factors that influence vaccination uptake. But, the current study’s use of ensemble machine learning models resulted in a significantly higher accuracy of 95.94% and identified the basic determinant factors that influence incomplete immunization among children under five age in Ethiopia.

Therefore, the studies by other researchers used various traditional statistical methods and simpler machine learning models, and generally achieved lower performance metrics compared to ensemble methods. The researchers were often limited by smaller datasets, not enough preprocessing techniques used, a limited model, clearly not analyzing the features with narrow age groups, and less advanced modeling techniques.

As a result, the voting ensemble methods achieved the best overall performance in this study, followed by the bagging ensemble model, and boosting ensemble methods also perform very well, with the stacking model being a strong method but slightly less effective compared to the others.

### Prediction factors of using bagging ME + XGBoost model

According to the results section, this study discovered that integrated features in the ensemble machine learning model perform very well in predicting the various factors for incomplete immunization. When analyzing factors influencing incomplete immunization, it’s essential to understand how various socio-demographic factors can affect immunization coverage.

The influence of marital status (9.01%) suggests the highest important factor in the prediction model of the Bagging ME + XGBoost algorithm, indicating that children from single-parent or non-married, divorced households are more likely to be incompletely immunized^24^^,26^^,28^^,29^^,30^^,31^ place of residence (8.64%), and employees, the second factor, is that living in rural areas or regions with limited healthcare^23,^^26^^,32^. Because infrastructure plays a critical role in incomplete immunization, underscoring the importance of improving healthcare access in these areas, and also the mother’s age (8.58%) is a strong predictor, where younger or older mothers may face barriers to accessing immunization services for their children^22,^^23^^,26^^,27^.

Accordingly, the marital status, place of residence, and mother’s characteristics are the primary factors determining whether a child completes their vaccination schedule. Also, the Bagging ME + XGBoost model highlights the importance of knowledge about immunization and media exposure, emphasizing that education status and healthcare outreach programs can have a significant positive impact. Furthermore, factors such as regional healthcare disparities and maternal education status underscore the need for localized interventions that take into account the specific needs of different communities.

Therefore, the voting ensemble method Bagging ME + XGBoost has the greatest results from all recent experiments and earlier investigations, achieving train accuracy, validation accuracy, and testing accuracy of 99.60%, 95.75%, and 95.94%, respectively.

### Study limitations

Despite the strong performance of ensemble models in this study, several challenges and limitations remain. First, the dataset exhibited an imbalance between incomplete and complete immunization cases, with incomplete immunization being more frequent. Although we applied techniques such as SMOTE (Synthetic Minority Over-sampling Technique) to address this imbalance, it is important to acknowledge the inherent trade-off between improving model accuracy and maintaining generalizability. Oversampling methods like SMOTE may lead to overfitting on synthetic examples, potentially limiting the model’s performance on truly unseen data. while ensemble models (bagging, boosting, voting, stacking) provide superior predictive accuracy, they are often more complex and less interpretable compared to simpler models such as logistic regression, artificial neural networks (ANN), support vector machines (SVM), and k-nearest neighbors (KNN). This complexity can hinder the translation of findings into actionable public health policies. Future research should explore the use of deep learning combined with explainable Artificial Intelligence (XAI) methods to improve both prediction and interpretability.

Also, the study relies on survey data that may be subject to social desirability bias, where respondents could overreport positive health behaviors, such as vaccinating their children, thus potentially inflating immunization coverage estimates. Additionally, recall bias may affect data accuracy because caregivers might not precisely remember vaccination details, introducing misclassification errors.

Finally, although the study utilizes a large, nationally representative dataset of Ethiopian children, the findings’ generalizability to other countries or regions with different sociocultural and health system contexts may be limited. Further studies were necessary to validate these models in diverse populations.

## Conclusion

This study summarizes the design of an incomplete immunization prediction model among Ethiopian children by applying ensemble learning algorithms. As far as Immunization remains one of the most cost-effective health interventions worldwide, crucial for preventing serious childhood diseases. To address gaps in immunization coverage for children aged 0 to 59 months, we applied several ensemble methods, including bagging, boosting, voting, and stacking. So, the required algorithms were used, bagging (Bagging meta-estimator, Random Forest), Boosting (GBoost, XGBoost, LGBM, AdaBoost, CatBoost), Voting (Bagging meta-estimator + Random Forest, GBoost + AdaBoost, LightGBM + CatBoost, Random Forest + LightGBM, Bagging ME + XBoost, AdaBoost + LightGBM, XGBoost + AdaBoost + LightGBM, LightGBM + GBoost + AdaBoost + XGBoost + CatBoost and stacking (as a base model XGBoost, CatBoost, RF, KNN, ANN, SVM and as a meta-model Logistic Regression) algorithms using nationally representative datasets from EDHS 2016 and EMDHS 2019. We implemented comprehensive preprocessing techniques such as data cleaning, reduction, and balancing through SMOTE, Tomek Link, and class weighting, which significantly enhanced model performance. Training and evaluation were performed using a split of 80% for training, 20% for testing data, and 5-fold cross-validation to ensure robustness. Among the tested models, the combination of Bagging Meta-Estimator and XGBoost achieved the highest predictive accuracy (95.94%), precision (97.00%), recall (94.81%), and F1 score (95.89%), cross-validation result of 95.75%, and visualizing through confusion matrix and AUC-ROC curve that shows the positive and the negative classes with a score of 96%.

The Bagging Meta-Estimator and XGBoost model identified key socio-demographic factors significantly associated with incomplete immunization, notably marital status, place of residence, and mother’s age, highlighting the influence of economic and social determinants on immunization coverage.

Therefore, this study demonstrates the effectiveness of ensemble machine learning approaches in predicting incomplete immunization, providing valuable insights for targeted interventions to improve child health outcomes in Ethiopia.

### Implications for public health policy

The findings of this study have important implications for public health policy, particularly in Ethiopia and similar low-resource settings. The high accuracy of the model (Bagging ME + XGBoost) in predicting incomplete immunization allows policymakers to:

#### Target high-risk groups

By identifying children most at risk of incomplete immunization based on factors like specific maritial status for single, divorced, or widowed mothers, who may face barriers to accessing healthcare services for their children.

#### Design better interventions

More accurately tailor immunization campaigns to address the factors contributing to incomplete immunization coverage.

#### Improve resource allocation

Direct resources toward regions or communities where incomplete immunization is more likely, thereby increasing overall vaccination rates.

### Recommendations

This study opens several avenues for future work:

#### Exploring new features

Future studies could incorporate more granular data and improve prediction models.

#### Refining models

Advanced algorithms like deep learning or hybrid models and Artificial Intelligence (AI) methods could be explored to see if further improvements in accuracy and interpretability can be achieved.

#### Cross-country comparisons

Expanding the research to include data from other countries could provide a broader perspective on global childhood immunization challenges.

## Appendix A: ethical approval


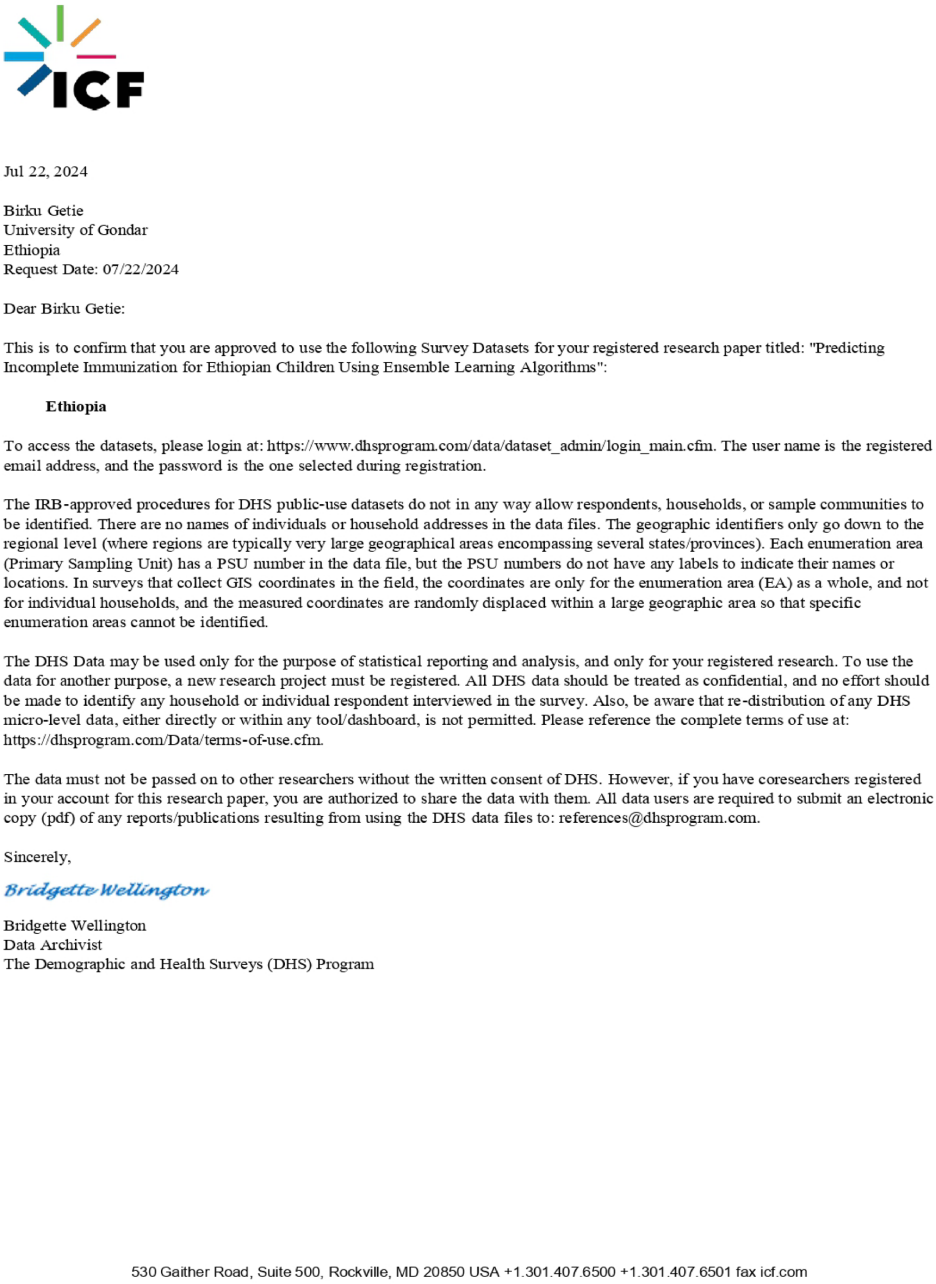


## Data Availability

The official database of the DHS program contains the dataset that was utilized and examined in this investigation (http://www.dhsprogram.com) upon formal request. An email is usually sent to confirm approval for access to the dataset.

## Abbreviations

AdaBoost: Adaptive Boosting
ANN: Artificial Neural Network
AUC-ROC: Area under Curve -Receiver Operating Characteristic
BCG: Bacille Calmette Guérin
CatBoost: Categorical Boosting
CSA: Central Statistical Agency
CV: Cross Validation
DHS: Demographic Health Survey
DPT: Diphtheria Pertussis Tetanus
EDA: Exploratory Data Analysis
EML: Ensemble Machine Learning
EPI: Expanded Program Immunization
FMoH: Federal Ministry of Health
GBoost: Gradient Boosting
ICF: International Coaching Federation
KNN: K-Nearest Neighbor
LGBM: Light Gradient Boosting Machine
LR: Logistics Regression
ME: Meta-Estimator
PART: Partial Decision Trees
PCV: Pneumococcal Conjugate Vaccine
RF: Random Forest
SVM: Support Vector Machine
UNICEF: United Nations International Children’s Emergency Fund
VPD: Vaccine-Preventable Diseases
WHO: World Health Organization
XGBoost: Extreme Gradient Boosting

## References

[1] Shattock, A. J. et al. Contribution of vaccination to improved survival and health: modelling 50 years of the expanded programme on immunization. *Lancet* **403**, 2307–2316 (2024). 38705159 10.1016/S0140-6736(24)00850-X PMC11140691

[2] WHO. Immunization - PAHO_WHO _ Pan American Health Organization. 1–2 at. (2023).

[3] Lindstrand, A., Cherian, T., Chang-Blanc, D., Feikin, D. & O’brien, K. L. The world of immunization: Achievements, Challenges, and strategic vision for the next decade. *J. Infect. Dis.* **224**, S452–S467 (2021). 34590130 10.1093/infdis/jiab284 PMC8482029

[4] Yadita, Z. S. & Ayehubizu, L. M. Full immunization coverage and associated factors among children aged 12–23 months in Somali Region, Eastern Ethiopia. *PLoS One* **16** (12), e0260258. 10.1371/journal.pone.0260258 (2021). 10.1371/journal.pone.0260258 PMC8651113 34874949

[5] Ndwandwe, D., Nnaji, C. A., Mashunye, T., Uthman, O. A. & Wiysonge, C. S. Incomplete vaccination and associated factors among children aged 12–23 months in South africa: an analysis of the South African demographic and health survey 2016. *Hum. Vaccines Immunother*. **17**, 247–254 (2021). 10.1080/21645515.2020.1791509 PMC7872074 32703070

[6] Zemariam, A. B. et al. Immunization coverage and its associated factors among children aged 12–23 months in ethiopia: an umbrella review of systematic review and meta-analysis studies. *PLoS One*. **19**, 1–18 (2024). 10.1371/journal.pone.0299384 PMC10919590 38451961

[7] Masebo, S. T. et al. Determinants of default from completion of child immunization among children aged 15–23 months in Kacha Bira district, Kembata Tembaro zone, South Ethiopia: A case–control study. *Front. Public Health* **12**, 1291495 (2024). 10.3389/fpubh.2024.1291495 PMC11074784 38716249

[8] Fetene, S. M. & Negash, W. D. Determinants of full immunization coverage among children 12–23 months of age from deviant mothers/caregivers in Ethiopia: A multilevel analysis using 2016 demographic and health survey. *Front. Public Health* **11**, 1085279 (2023). 10.3389/fpubh.2023.1085279 PMC10011448 36926180

[9] Kebede Kassaw, A. A. et al. Spatial distribution and machine learning prediction of sexually transmitted infections and associated factors among sexually active men and women in Ethiopia, evidence from EDHS 2016. *BMC Infect. Dis.* **23**, 1–16 (2023). 36690950 10.1186/s12879-023-07987-6 PMC9872341

[10] Sako, S., Gilano, G. & Hailegebreal, S. Determinants of childhood vaccination among children aged 12–23 months in ethiopia: a community-based cross-sectional study. *BMJ Open.* **13**, 1–9 (2023). 10.1136/bmjopen-2022-069278 PMC10008449 36889833

[11] Shroff, Z. C. et al. Decision-maker-led implementation research on immunization: learning from low- and middle-income countries. *Heal Res. Policy Syst.* **19**, 1–5 (2021). 10.1186/s12961-021-00720-2 PMC8356372 34380520

[12] Gelagay, A. A. et al. Complete childhood vaccination and associated factors among children aged 12–23 months in Dabat demographic and health survey site, Ethiopia, 2022. *BMC Public. Health*. **23**, 1–9 (2023). 37131146 10.1186/s12889-023-15681-0 PMC10152426

[13] Melaku, M. S., Nigatu, A. M. & Mewosha, W. Z. Spatial distribution of incomplete immunization among under-five children in ethiopia: Evidence from 2005, 2011, and 2016 Ethiopian demographic and health survey data. *Arch. Public Health* **78**, 1–13 (2020). 10.1186/s12889-020-09461-3 PMC7487875 32891120

[14] Mahajan, P., Uddin, S., Hajati, F. & Moni, M. A. Ensemble learning for disease prediction: A review. *Healthcare* **11** (12), 1808. 10.3390/healthcare11121808 (2023). 10.3390/healthcare11121808 PMC10298658 37372925

[15] Boardman, J., Biron, K. & Rimbey, R. Mitigating the Effects of Class Imbalance Using Smote and Tomek Link Undersampling in SAS ^®^. Proc. SAS Glob. Forum 2018 Conf. 1–20 (2018).

[16] Budu, E. & Bagging Boosting, and Stacking in Machine Learning | Baeldung on Computer Science. at (2023). https://www.baeldung.com/cs/bagging-boosting-stacking-ml-ensemble-models

[17] n.d. Evaluation Metrics in Machine Learning. Geeksforgeeks at (2024). https://www.geeksforgeeks.org/metrics-for-machine-learning-model/

[18] EPI. Expanded Program on Immunization (EPI) | MINISTRY OF HEALTH - Ethiopia. at. (2021).

[19] Immunization Service Desk _ MINISTRY OF HEALTH - Ethiopia.

[20] WHO. Immunization Agenda 2030. Who 1–58. (2022).

[21] Asresie, M. B., Dagnew, G. W. & Bekele, Y. A. Changes in immunization coverage and contributing factors among children aged 12–23 months from 2000 to 2019, ethiopia: multivariate decomposition analysis. *PLoS One*. **18**, 1–15 (2023). 10.1371/journal.pone.0291499 PMC10499235 37703252

[22] Debie, A., Lakew, A. M., Tamirat, K. S., Amare, G. & Tesema, G. A. Complete vaccination service utilization inequalities among children aged 12–23 months in ethiopia: A multivariate decomposition analysis. *Int. J. Equity Health*. **19**, 1–16 (2020). 10.1186/s12939-020-01166-8 PMC7218567 32398089

[23] Bago, B. J., Terefe, W. & Mirutse, G. Individual and community level factors associated with defaulting of immunization among 12–59 months children in ethiopia: multilevel modeling analysis using 2011Ethiopia demographic and health survey. *Curr. Pediatr. Res.* **22**, 95–110 (2018).

[24] Gebremariam, M. et al. Determinants of incomplete vaccination among children age 12–23 months in Southwest ethiopia: A Case-Control study. *OMO Int. J. Sci.* **3**, 56–69 (2020).

[25] Mebrat, A., Dube, L., Kebede, A. & Aweke, Z. Determinants of incomplete childhood vaccination among children aged 12–23 months in Gambela Region, Southwest ethiopia: A case control study. *Ethiop. J. Health Sci.* **31**, 63–72 (2021). 34158753 10.4314/ejhs.v31i1.8 PMC8188105

[26] Muluye, M. et al. Partial vaccination and associated factors among children aged 12–23 months in Eastern Ethiopia. *BMC Pediatr.* **22**, 1–10 (2022). 35550040 10.1186/s12887-022-03320-3 PMC9097114

[27] Dana, A. D., Kebede, D. L. & Betru, K. T. Prevalence of incomplete vaccination and associated factors among children aged 24–35 months in Dale woreda, Sidama region, Ethiopia. *Ethiop. J. Health Sci.* **2** (2), 136–149. 10.82127/7ga5dh29 (2022).

[28] Desalew, A., Semahegn, A., Birhanu, S. & Tesfaye, G. Incomplete vaccination and its predictors among children in Ethiopia: A systematic review and meta‑analysis. *Glob. Pediatr. Health* **7**, 2333794X20968681. 10.1177/2333794X20968681 (2020). 10.1177/2333794X20968681 PMC7675896 33241080

[29] Boke, M. M., Tenaw, G., Berhe, N. M. & Tiruneh, W. K. Determinants of incomplete childhood immunization among children aged 12–23 months in Dabat district, Northwest ethiopia: unmatched case-control study. *PLoS One*. **17**, 1–13 (2022). 10.1371/journal.pone.0274501 PMC9584397 36264780

[30] Tesema, G. A., Tessema, Z. T., Tamirat, K. S. & Teshale, A. B. Complete basic childhood vaccination and associated factors among children aged 12–23 months in East africa: a multilevel analysis of recent demographic and health surveys. *BMC Public. Health*. **20**, 1–14 (2020). 33256701 10.1186/s12889-020-09965-y PMC7708214

[31] Dheresa, M. et al. Child vaccination coverage, trends and predictors in Eastern ethiopia: implications for sustainable development goals. *J. Multidiscip Healthc.* **14**, 2657–2667 (2021). 34584421 10.2147/JMDH.S325705 PMC8464587

[32] Faisal, S. et al. Modeling the Factors Associated with Incomplete Immunization among Children. Math. Probl. Eng. (2022). (2022).

[33] Childhood Immunization Schedule. Vaccines By Age. at https://my.clevelandclinic.org/health/articles/11288-childhood-immunization-schedule

[34] Negussie, A., Kassahun, W., Assegid, S. & Hagan, A. K. Factors associated with incomplete childhood immunization in Arbegona district, Southern ethiopia: A case-control study. *BMC Public. Health*. **16**, 1–9 (2016). 26757893 10.1186/s12889-015-2678-1 PMC4711011

[35] Emiru, T. D., Debrework, T., Marye, G. & Chalie, M. T. *Assessment of Vaccination Coverage and Associated Factors among Children Aged 12–23 Months in Debre Tabor Town, North West Ethiopia* (Research Sq, 2021).

[36] Nour, T. Y. et al. Predictors of immunization coverage among 12–23-month-old children in ethiopia: systematic review and meta-analysis. *BMC Public. Health*. **20**, 1–19 (2020). 33243208 10.1186/s12889-020-09890-0 PMC7689978

[37] Demsash, A. W. et al. Machine learning algorithms’ application to predict childhood vaccination among children aged 12–23 months in ethiopia: evidence 2016 Ethiopian demographic and health survey dataset. *PLoS One*. **18**, 1–22 (2023). 10.1371/journal.pone.0288867 PMC10584162 37851705

[38] Chandir, S. et al. Using predictive analytics to identify children at high risk of defaulting from a routine immunization program: feasibility study. *JMIR Public. Heal Surveill*. **4**, 1–12 (2018). 10.2196/publichealth.9681 PMC6231754 30181112

[39] Bitew, F. H., Nyarko, S. H., Potter, L. & Sparks, C. S. Machine learning approach for predicting under-five mortality determinants in Ethiopia: Evidence from the 2016 Ethiopian Demographic and Health Survey. *Genus* **76**, 37. 10.1186/s41118-020-00106-2 (2020).

[40] Tadese, Z. B., Nigatu, A. M., Yehuala, T. Z. & Sebastian, Y. Prediction of incomplete immunization among under-five children in East Africa from recent demographic and health surveys: a machine learning approach. *Sci. Rep.* **14**, 1–14 (2024). 38773175 10.1038/s41598-024-62641-8 PMC11109113

[41] Exploratory Data Analysis in Machine Learning for Data Science. at https://www.linkedin.com/pulse/exploratory-data-analysis-machine-learning-science-aritra-pain

[42] Fontana, R., Molena, A., Pegoraro, L. & Salmaso, L. Design of experiments and machine learning with application to industrial experiments. *Stat. Pap*. **64**, 1251–1274 (2023).

[43] Muntean, M. & Militaru, F. D. Design science research framework for performance analysis using machine learning techniques. *Electronics* **11** (16), 2504 (2022).

[44] Novogroder, I. Data Preprocessing in Machine Learning: Steps & Best Practices. at https://lakefs.io/blog/data-preprocessing-in-machine-le&#8230.

[45] Skillfloor The Role of Data Preprocessing in Machine Learning | by Skillfloor | Medium. at (2023). https://skillfloor.medium.com/the-role-of-data-preprocessing-in-machine-learning-d9ad9db54d49

[46] The Pecan Team. Data Preparation for Machine Learning: The Ultimate Guide | Pecan AI. at (2023). https://www.pecan.ai/blog/data-preparation-for-machine-learning/

[47] CSA. Demographic and Health Survey | UNICEF Ethiopia. at (2018). https://www.unicef.org/ethiopia/reports/demographic-and-health-survey

[48] Ethiopia Mini Demographic and Health Survey 2019 – Healthy Newborn Network. at https://www.healthynewbornnetwork.org/resource/ethiopia-mini-demographic-and-health-survey-2019/

[49] Abegaz, M. Y., Seid, A., Awol, S. M. & Hassen, S. L. Determinants of incomplete child vaccination among mothers of children aged 12–23 months in Worebabo district, ethiopia: unmatched case-control study. *PLOS Glob Public. Heal*. **3**, 1–15 (2023). 10.1371/journal.pgph.0002088 PMC10431650 37585408

[50] Yismaw, A. E., Assimamaw, N. T., Bayu, N. H. & Mekonen, S. S. Incomplete childhood vaccination and associated factors among children aged 12–23 months in Gondar City administration, Northwest, Ethiopia 2018. *BMC Res. Notes*. **12**, 1–7 (2019). 31036071 10.1186/s13104-019-4276-2 PMC6489187

[51] Damete, D. D. The study on vaccination coverage among under-five children in Ethiopia revealed concerning levels of inequality and low overall coverage rates, with only 55.0% of children receiving vaccinations. Mini Demographic and Health Survey. 1–15 (2023). (2019).

[52] Amit Diwan. The K-Fold Cross-Validation in Machine Learning. at https://www.knowledgehut.com/blog/data-science/k-fold-cross-validation-in-ml

[53] Brownlee, J. A. Gentle Introduction to Ensemble Learning Algorithms - MachineLearningMastery.com. Machine Learning Mastery 1 at (2021). https://machinelearningmastery.com/tour-of-ensemble-learning-algorithms/

[54] Ali Awan, A. A. Guide to Bagging in Machine Learning: Ensemble Method to Reduce Variance and Improve Accuracy. DataCamp at (2023). https://www.datacamp.com/tutorial/what-bagging-in-machine-learning-a-guide-with-examples

[55] Avijeet, B. What is Bagging in Machine Learning, And How to Perform Bagging. SimpliLearn at (2023). https://www.simplilearn.com/tutorials/machine-learning-tutorial/bagging-in-machine-learning

[56] Keylabs Random Forest: Ensemble Learning Technique. Keylabs: latest news and updates at (2024). https://keylabs.ai/blog/random-forest-ensemble-learning-technique/

[57] Boosting Algorithms In ML: GBM, XGboost, LightBoost, CatBoost. at https://www.analyticsvidhya.com/blog/2020/02/4-boosting-algorithms-machine-learning/

[58] Boosting Algorithms. AdaBoost, Gradient Boosting, and XGBoost | HackerNoon. at https://hackernoon.com/boosting-algorithms-adaboost-gradient-boosting-and-xgboost-f74991cad38c

[59] CFI Team. Ensemble Methods - Overview, Categories, Main Types. at (2023). https://corporatefinanceinstitute.com/resources/data-science/ensemble-methods/

[60] Navlani, A. AdaBoost Classifier Algorithms using Python Sklearn Tutorial | DataCamp. at (2018). https://www.datacamp.com/tutorial/adaboost-classifier-python

[61] Voting in Machine Learning - GeeksforGeeks.

[62] Voting Classifier - GeeksforGeeks. at https://www.geeksforgeeks.org/voting-classifier/

[63] Working, K. I. T., Series, P. & Voting A machine learning approach. Voting : a machine learning approach by Dávid Burka, Clemens Puppe, László Szepesváry. (2020).

[64] Brownlee, J. Stacking ensemble machine learning with Python - machineLearningMastery.com. Machine Learning Mastery at (2021). https://machinelearningmastery.com/stacking-ensemble-machine-learning-with-python/

[65] Stacking to Improve Model Performance. A Comprehensive Guide on Ensemble Learning in Python | by Brijesh Soni | Medium. at https://medium.com/@brijesh_soni/stacking-to-improve-model-performance-a-comprehensive-guide-on-ensemble-learning-in-python-9ed53c93ce28

[66] stacking. Stacking in Machine Learning - GeeksforGeeks. at (2023). https://www.geeksforgeeks.org/stacking-in-machine-learning-/

[67] Artificial Neural Networks in. Machine Learning | Tirendaz AI | MLearning.ai. at https://medium.com/mlearning-ai/artificial-neural-networks-in-machine-learning-fa653d74b1a1

[68] DataFlair Artificial Neural Networks for Machine Learning – Every aspect you need to know about. at (2018). https://data-flair.training/blogs/artificial-neural-networks-for-machine-learning/

[69] aswathisasidharan. Support Vector Machine (SVM) Algorithm - GeeksforGeeks. GeeksforGeeks at (2024). https://www.geeksforgeeks.org/support-vector-machine-algorithm/

[70] K-Nearest. Neighbor(KNN) Algorithm - GeeksforGeeks.

[71] Geeks Group. Supervised Machine Learning | GeeksforGeeks. at (2025). https://www.geeksforgeeks.org/supervised-machine-learning/

[72] Performance Metrics in Machine Learning - Javatpoint. at (2022). https://www.javatpoint.com/performance-metrics-in-machine-learning

[73] Geeksforgeeks.org. Confusion Matrix in Machine Learning - GeeksforGeeks. at (2020). https://www.geeksforgeeks.org/confusion-matrix-machine-learning/

[74] Optimal Threshold for Imbalanced Classification. | by Audhi Aprilliant | Towards Data Science. at https://towardsdatascience.com/optimal-threshold-for-imbalanced-classification-5884e870c293

[75] Shrivastava, I. Handling Class Imbalance by Introducing Sample Weighting in the Loss Function | by Ishan Shrivastava | GumGum Tech Blog | Medium. at (2020). https://medium.com/gumgum-tech/handling-class-imbalance-by-introducing-sample-weighting-in-the-loss-function-3bdebd8203b4

[76] Cheong, Q., Au-Yeung, M., Quon, S., Concepcion, K. & Kong, J. D. Predictive modeling of vaccination uptake in US counties: A machine learning-based approach. *J. Med. Internet Res.* **23**, 1–10 (2021). 10.2196/33231 PMC8623305 34751650

